# Social encounter profiles of greater Melbourne residents, by location – a telephone survey

**DOI:** 10.1186/s12879-015-1237-9

**Published:** 2015-11-02

**Authors:** David A. Rolls, Nicholas L. Geard, Deborah J. Warr, Paula M. Nathan, Garry L. Robins, Philippa E. Pattison, James M. McCaw, Jodie McVernon

**Affiliations:** Melbourne School of Psychological Sciences, The University of Melbourne, Melbourne, Australia; Modelling and Simulation Unit, Centre for Epidemiology and Biostatistics, Melbourne School of Population and Global Health, The University of Melbourne, Melbourne, Australia; McCaughey VicHealth Community Wellbeing Unit, Melbourne School of Population and Global Health, The University of Melbourne, Melbourne, Australia; School of Mathematics and Statistics, The University of Melbourne, Melbourne, Australia; Modelling and Simulation Unit, Infection and Immunity Theme, Murdoch Childrens Research Institute, Parkville, Australia

**Keywords:** Social networks, Socioeconomic factors, Population characteristics

## Abstract

**Background:**

Models of infectious disease increasingly seek to incorporate heterogeneity of social interactions to more accurately characterise disease spread. We measured attributes of social encounters in two areas of Greater Melbourne, using a telephone survey.

**Methods:**

A market research company conducted computer assisted telephone interviews (CATIs) of residents of the Boroondara and Hume local government areas (LGAs), which differ markedly in ethnic composition, age distribution and household socioeconomic status. Survey items included household demographic and socio-economic characteristics, locations visited during the preceding day, and social encounters involving two-way conversation or physical contact. Descriptive summary measures were reported and compared using weight adjusted Wald tests of group means.

**Results:**

The overall response rate was 37.6 %, higher in Boroondara [*n* = 650, (46 %)] than Hume [*n* = 657 (32 %)]. Survey conduct through the CATI format was challenging, with implications for representativeness and data quality. Marked heterogeneity of encounter profiles was observed across age groups and locations. Household settings afforded greatest opportunity for prolonged close contact, particularly between women and children. Young and middle-aged men reported more age-assortative mixing, often with non-household members. Preliminary comparisons between LGAs suggested that mixing occurred in different settings. In addition, gender differences in mixing with household and non-household members, including strangers, were observed by area.

**Conclusions:**

Survey administration by CATI was challenging, but rich data were obtained, revealing marked heterogeneity of social behaviour. Marked dissimilarities in patterns of prolonged close mixing were demonstrated by gender. In addition, preliminary observations of between-area differences in socialisation warrant further evaluation.

**Electronic supplementary material:**

The online version of this article (doi:10.1186/s12879-015-1237-9) contains supplementary material, which is available to authorized users.

## Background

Models of infectious disease epidemiology are becoming ever more complex in their construction, in recognition of the importance of heterogeneity of individuals and their social interactions to transmission of infection [1]. Such models have been informed by the increasing acquisition of data in a range of studies, describing patterns of human interaction at the level of age group [2], within social networks [3], or related to movements in geographical space and time [4].

In earlier work, we piloted methods to acquire data on social encounters of individuals within the locations visited over the course of several days, comparing anticipated contacts with prospective records entered either in a paper diary (modified from European study instruments) or portable electronic device [5, 6]. While that study provided rich and detailed information, the scope of data collection was necessarily limited by budgetary and logistic constraints associated with the requirement for face-to-face recruitment and training, making detailed characterization of large population samples challenging.

In this project, we are seeking to document the influence of individual and area-level factors on social encounter profiles, which have been qualitatively observed to influence perceived social network characteristics [7]. In particular, individuals from less advantaged neighbourhoods have been described as likely to have close local networks, and fewer ‘bridging’ ties beyond their immediate area in comparison with counterparts in socio-economically advantaged areas who are more likely to have broadly distributed connections [7]. This project seeks to define in more detail the attributes of social encounters, beyond degree distribution, that might quantifiably capture such differences.

We recruited residents of two local government areas of metropolitan Melbourne (the state capital of Victoria, Australia) that differ markedly in terms of demographics and socio-economic status as ‘proof of concept’ of such influence. In order to reach as large and broadly representative a sample as possible, we employed telephone-based survey methods for recruitment and collection of encounter data. This paper reports on our experience of this survey approach, and initial findings summarising encounters in the two regions of interest.

## Methods

### Study population

**Fig. 1:**
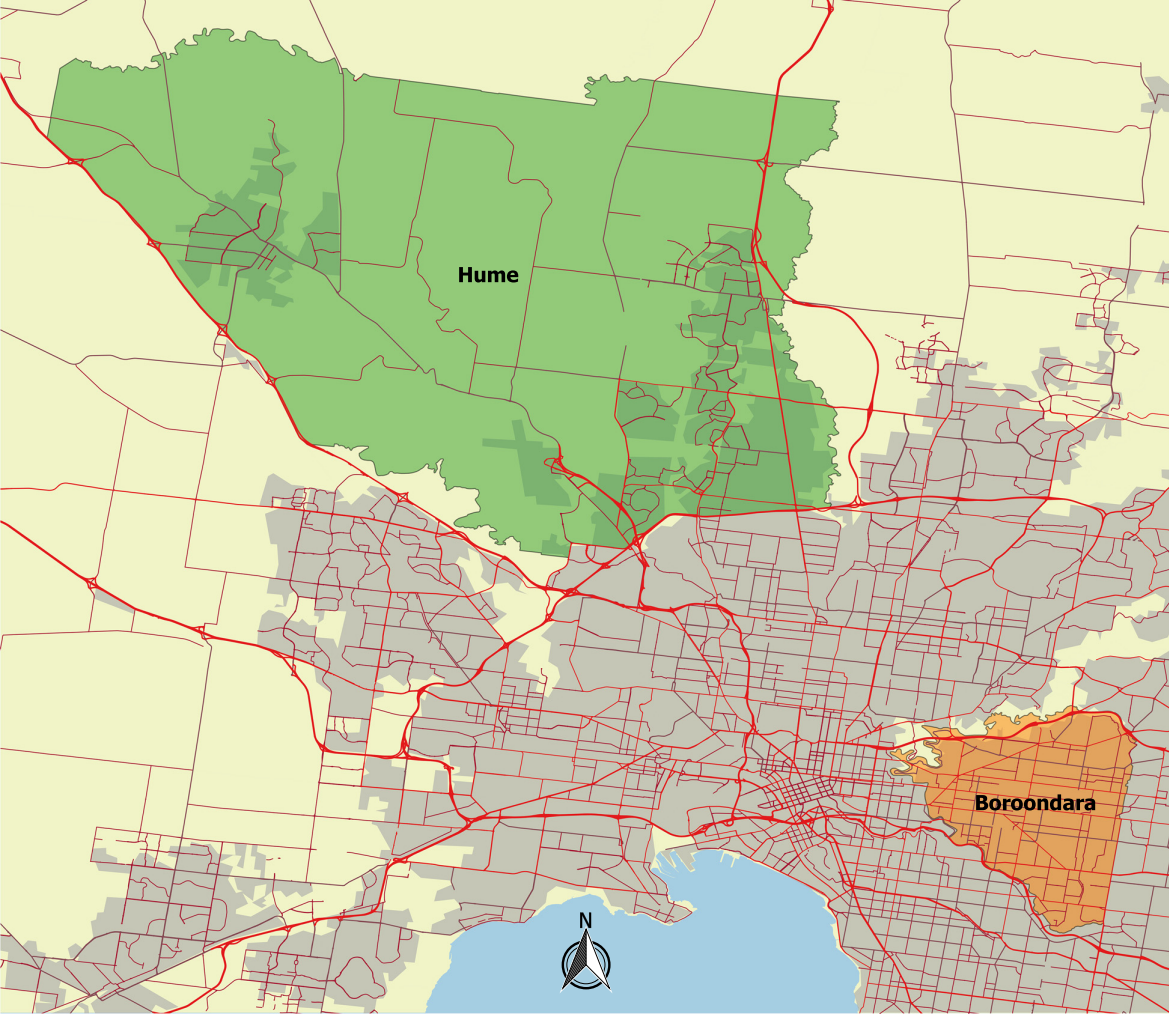
Map showing geographical extent of Hume and Boroondara Local Government Areas, within the context of Greater Melbourne. The map was generated with QGIS software using data from the Australian Bureau of Statistics [36, 37]. *Red lines* depict the road network [12], and *shading* denotes built-up areas [38]

The study recruited participants from two local government areas (LGAs) in greater Melbourne between January and April 2013 (Fig. 1). Each area had a similar population size but markedly different population characteristics, as outlined below.

*Hume* is a growing fringe municipality 20 kms from Melbourne’s central business district (CBD), retaining a rural character in its North. 43 % of its population was born overseas with the most frequent countries of origin being Turkey (5 %) and Iraq (5 %), and 31 % of residents were less than 18 years of age according to the 2011 census [8]. Median household income for families with children was around AUD$1,300 per week [8]. In contrast, *Boroondara* incorporates a number of established inner-eastern suburbs, 5 kms east of the CBD, bounded by rivers and parkland. While 34 % of its residents were born overseas, the most common countries of origin were China (5 %) and the United Kingdom (3 %), and only 21 % were less than 18 years old in 2011 [9]. Median household income for families with dependent children at that time exceeded AUD$2,500 per week [9]. Further details of the demographic and socioeconomic characteristics of each LGA can be found in the Additional file 1: Cleaning, Geocoding and Weighting.

### Survey methods

A market research company was contracted to conduct computer assisted telephone interviews (CATI) of 1000 respondents in each LGA to characterize social encounter profiles in locations visited over the course of a single day. Random digit dialling within local telephone exchanges was used to identify the sample, in order to avoid selection bias for longer-term resident individuals associated with the use of listed numbers (as short term tenants may change land lines frequently), as well as residents choosing to withhold their details from public listing. Unfortunately, individuals with only mobile phone or voice over internet protocol telephone access could not be included in the frame as it was not possible to assign such numbers to a residential location within the LGA boundaries*.* Respondents in Hume were given the option of completing the survey in either English or Turkish, given the high proportion (5 %) of Turkish born residents in that LGA, but resources were not available to offer interviews in other languages.

During a telephone interview (approximately 20–30 min in length), participants were asked to describe the basic demographic characteristics of their household including the number, age and occupational status of family members and key indicators of household economic status, including housing tenure. They were then asked to sequentially list all locations visited and movements between those locations on the previous day, including the type of location (e.g., home, work, retail, private transport) and when they left the location. They were also asked to list all social encounters with an individual or defined group (e.g., school class, workplace contacts, church congregation) for ease of reporting in each location. Encounters were defined as a two-way face-to-face conversation of more than three words or any physical contact. All interviews with participants were conducted by telephone and entered into an electronic form by employees of the market research company. Survey instruments are provided in Additional file 2: Questionnaire and Additional file 3: Contact diary.

### Data preparation

Addresses of visited locations were checked for accuracy and completeness, and corrected to a standard format. Where necessary, place name descriptions were assigned street addresses with reference to corporate websites (retail locations) and/or publicly available searchable mapping tools. Time data were similarly checked for logical order and consistency, and calculated in relation to expected travel time between locations as necessary for confirmation. Further details of standard procedures followed for data cleaning are provided in Additional file 1 (Section 2 – Address Accuracy and Cleaning and Section 3 – Time Consistency and Cleaning).

### Geocoding

Addresses were geocoded using a mix of API queries including Bing Maps [10], Mapquest [11] and OpenStreetMaps [12], and manually via the Google Maps website [13]. MATLAB 7.14 was used to script all work. Rules for geocoding, location matching and confirmation of accuracy are described in more detail in Additional file 1 (Section 4 - Geocoding).

### Weighting

Demographic and socio-economic characteristics of the LGAs under study were obtained from the Australian Bureau of Statistics 2011 census using publicly available methods [14]. Iterative proportional fitting (i.e., raking) was used to determine sampling weights to reduce the effects of sample bias. Raking weights were computed and applied to the survey data using STATA 10 with reference to the following population descriptors.

1. Joint distribution of five age categories and gender (10 categories);
2. Marital status (coupled, never married, other);
3. Australian born (yes/no);
4. Household type (couple with children, couple without children, one parent family, other family, lone person household, other household);
5. Household size (1, 2, 3, 4, 5, 6+);
6. Home ownership (owned outright, owned with a mortgage, renting, other, missing).

Large weights were truncated at 7 (Boroondara) and 6 (Hume) to remove extreme weights. These cutoffs were the smallest values for which goodness-of-fit tests comparing raked variables with census totals did not reject at the 5 % level. Additional details and justification of the weighting procedure are available in Additional file 1 (Section 5 – Biased Sampling, Raking and Sampling Weights). In particular, goodness-of-fit results comparing the sample with the 2011 census both before and after raking are shown in Table S7 of Additional file 1.

### Analysis of social encounters

Summary measures of recorded encounters with individuals and groups were characterised for comparison with our own and other previous studies, including separate description of the subset involving any physical contact as a proxy measure of intensity. The number and duration of contacts was reported by location type. Interactions within and between age groups were considered separately for men and women. Social heterogeneity was further assessed, by differentiating between contacts with household members, known individuals and strangers. The influence of household size on the number of encounters within and beyond the household unit was considered. These various measures were tabulated by LGA of residence, and differences between regions assessed using weight-adjusted Wald tests of the difference between group means.

### Ethical approvals

The study protocol was approved by the University of Melbourne Human Research Ethics Committee (Ethics ID 1238477). Participants gave verbal informed consent to study participation prior to administration of the telephone questionnaire.

## Results

### Study population

A total of 25,406 calls were made to 8567 numbers, of which 7129 were currently connected telephones in residential households (2755 of 3398 in Boroondara, 4374 of 5169 in Hume). Contact was made with an individual in 4580 of these households (1827 in Boroondara and 2753 in Hume), with a further 683 proving ineligible due to geographical location (out of area), communication difficulties or absence of an adult present at the time of call. Communication difficulties reported included language difficulty (*n* = 28) or other physical limitation such as hearing impairment or age (*n* = 131). Of the remaining 3897 eligible contacts, 1307 (650 in Boroondara, 657 in Hume) completed the survey, with 12 interviews conducted in Turkish. Responses were spread across days of the week such that the numbers are fairly even across weekdays (range: 191—211) and separately across weekends (range: 156—157). Response rates according to the ‘all contacts’ denominator were 36 % (650/1827) in Boroondara and 24 % (657/2753) in Hume. When calculated as all completed interviews over a denominator comprising completions, refusals and break-offs, the response rate overall was 37.6 %, remaining higher in Boroondara (46 %) than Hume (32 %).

While the final number in the sample was less than our initial target of 2000, a pragmatic decision was made to cease recruitment at 1307, given greater than anticipated time requirements per participant and finite budgetary constraints. Implications of study complexity related to the telephone interview format for missing and incomplete data are described in more detail below.

### Weighting

Compared with 2011 Australian census data, characteristics of the sample differed significantly from those of the areas surveyed across a range of demographic and socio-economic factors in both Hume and Boroondara. Important differences included an over-representation of individuals who were aged over 50 years, female, Australian born, English speaking, educated to completion of secondary school, and married. In keeping with these characteristics, smaller households were over-represented. The anticipated bias towards longer resident individuals was also observed. Given this disparity, weighted results are presented for all aggregated data, in order to present results more likely to be representative of the populations under study. For more details, see Sections 5 *Biased sampling, raking and sampling weights* and 6 *Tables demonstrating bias in CATI data* of Additional file 1.

### Characteristics of encounters, by location

**Fig. 2:**
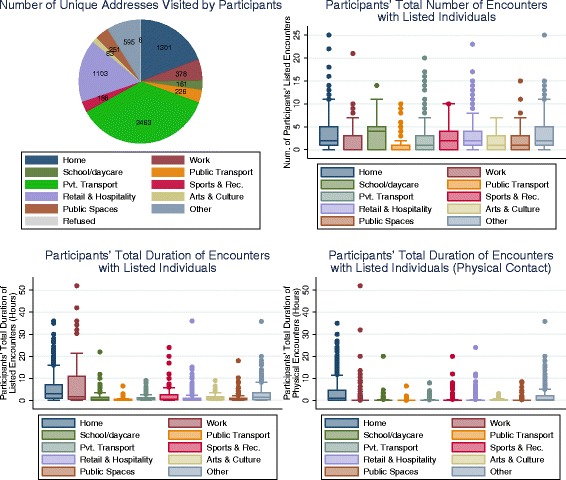
Characteristics of locations and encounters by location type. Summaries of locations and encounters from one study day for each participant, reported separately for each location type. The number of unique addresses visited by participants (*top left panel*). Boxplot for participants' total (weighted) number of encounters with listed individuals, by location (*top right panel*). Boxplot for participants' total (weighted) duration of encounters with listed individuals, by location (*bottom left panel*). Boxplot for participants' total duration of encounters involving physical contact with listed individuals, by location (*bottom right panel*). For all boxplots, boxes denote the interquartile range, interior lines shown the median and the whiskers show adjacent values. Across the boxplots, marked heterogeneity is apparent in several location types. For total duration of contact, Work (and Home to a lesser degree) is the dominant location type. For total duration of physical contact, home is the dominant location type. Boxplots use raking weights to reduce the effects of sample bias. For each location type, participants with no time spent at that location type do not contribute to those results

Unique addresses visited by participants over the course of the survey day were categorised and distributed as shown in Fig. 2 (top left panel). All but six participants spent some time at their usual home, with retail/hospitality, and private transport being the next most common types of designated settings. Approximately one tenth of locations were assigned as ‘other’, of which about half were non-participant private homes, with the remainder comprising places such as medical centres and facilities, and places of worship. The weighted number of total listed encounters by location is reported in Fig. 2 (top right panel) (and Additional file 4: Figure S1 for physical contact). These data demonstrate the high median number of contacts made in school and daycare settings, but also the marked heterogeneity in encounter profiles within several location types including home, private transport, retail/hospitality and ‘other’. Figure 2 (bottom left panel) highlights the greater length of interactions with workplace colleagues over the course of a working day, relative to those in most other domains. Figure 2 (bottom right panel) similarly reports duration of encounters, but restricted to those involving any physical contact. This last figure strongly reasserts the importance of household settings in providing opportunities for close-contact transmission of infection.

### Number and duration of social encounters, by age and gender

**Fig. 3:**
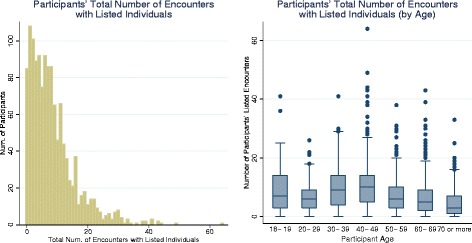
Participants’ total number of encounters with listed individuals. Histogram for participants’ total (unweighted) number of encounters with listed individuals (*left panel*). Boxplot for participants’ total (weighted) number of encounters with listed individuals by participant age (*right panel*). Heterogeneity is apparent across participants and across age groups. In general, participants' number of encounters declines with age. The boxplot uses raking weights to reduce the effects of sample bias

Figure 3 (left panel) reports the unweighted total number of listed encounters for each participant in the survey, with a mean of 8.4 (95 % CI: 7.9–8.8) and median of 7. The weighted total number of listed encounters for each participant across all participants (mean 8.5, 95 % CI: 7.9–9.1) and across days of the week (mean range 8.0–9.3, median range 6–8) was similar. In addition, the distinction between weekend and weekday is not a significant predictor for the number of listed encounters (*p* = 0.9). Figure 3 (right panel) shows a boxplot of the weighted number of all encounters by age group. The highest reported number of median contacts was among individuals aged between 30 and 49 years. This trend was more evident for episodes involving physical contact (Additional file 4: Figures S2 and S3).

**Fig. 4:**
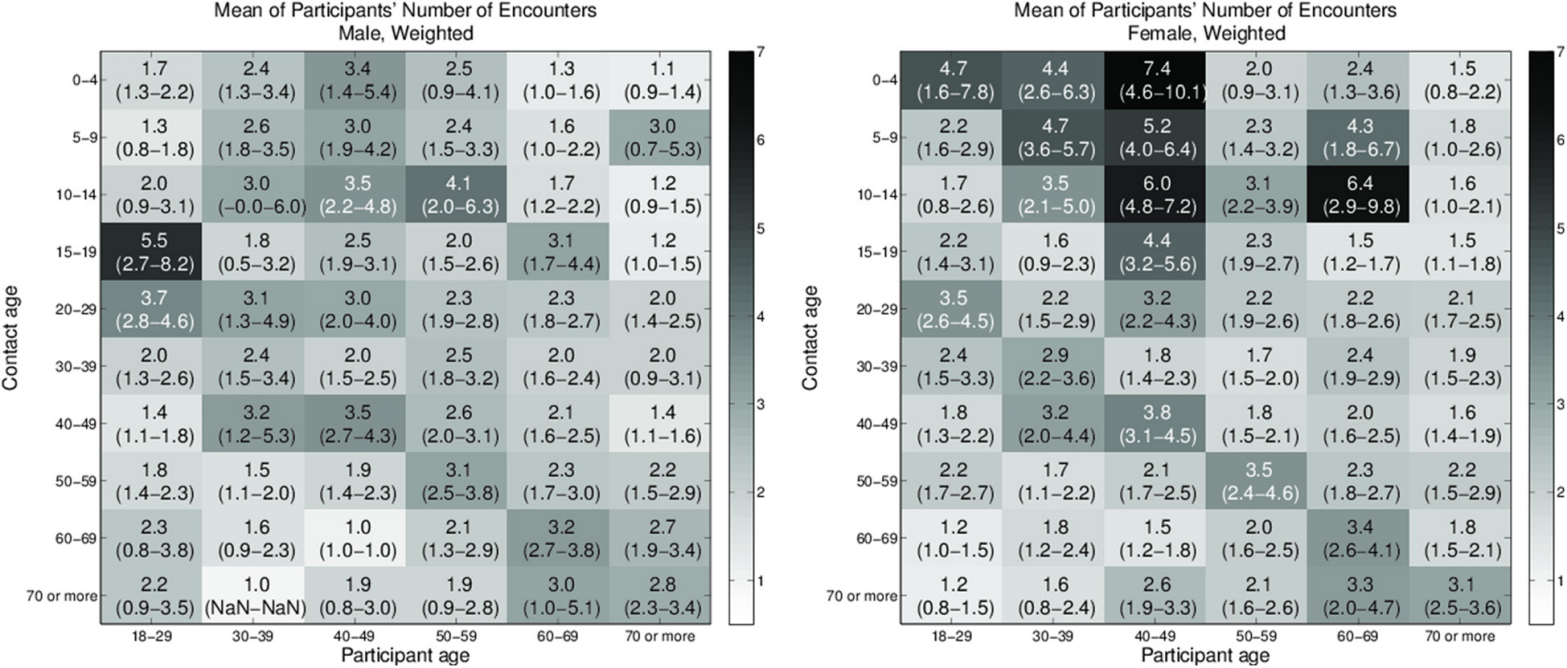
Age-based mixing matrices for participants’ total number of encounters with listed individuals. Values are the mean of participants’ total (weighted) number of encounters with listed individuals by age of participant and contact for male participants (*left panel*), and female participants (*right panel*). 95 % confidence intervals are shown in parentheses. Darker shading indicates larger values. Age-based assortative mixing is evident for both genders. Women notably have more numerous contacts with children less than 15 years old. Males 18–29 have the most numerous contacts within age groups. Raking weights are used to reduce the effects of sample bias

Respondents frequently reported more than one encounter with the same individual over a 24 h period (Additional file 4: Figure S4). The weighted mean number of contacts (with 95 % confidence intervals) between participants and uniquely nominated individuals is shown in Fig. 4. Results are reported across six participant age categories and ten contact age categories, for male (left panel) and female (right panel) respondents. The number of participants contributing to each cell varies, and both weighted and unweighted counts for each gender can be found in the supporting information (Additional file 4: Tables S1–S4 for all encounters and Tables S5–S8 for physical encounters). While a generally assortative pattern of mixing is observed, we also note that females record many more encounters with children less than 15 years of age than males. Males aged 18–29, on the other hand, have the highest recorded within-age group interactions. Similar trends are observed for episodes involving any physical contact (Additional file 4: Figures S7 and S8).

**Fig. 5:**
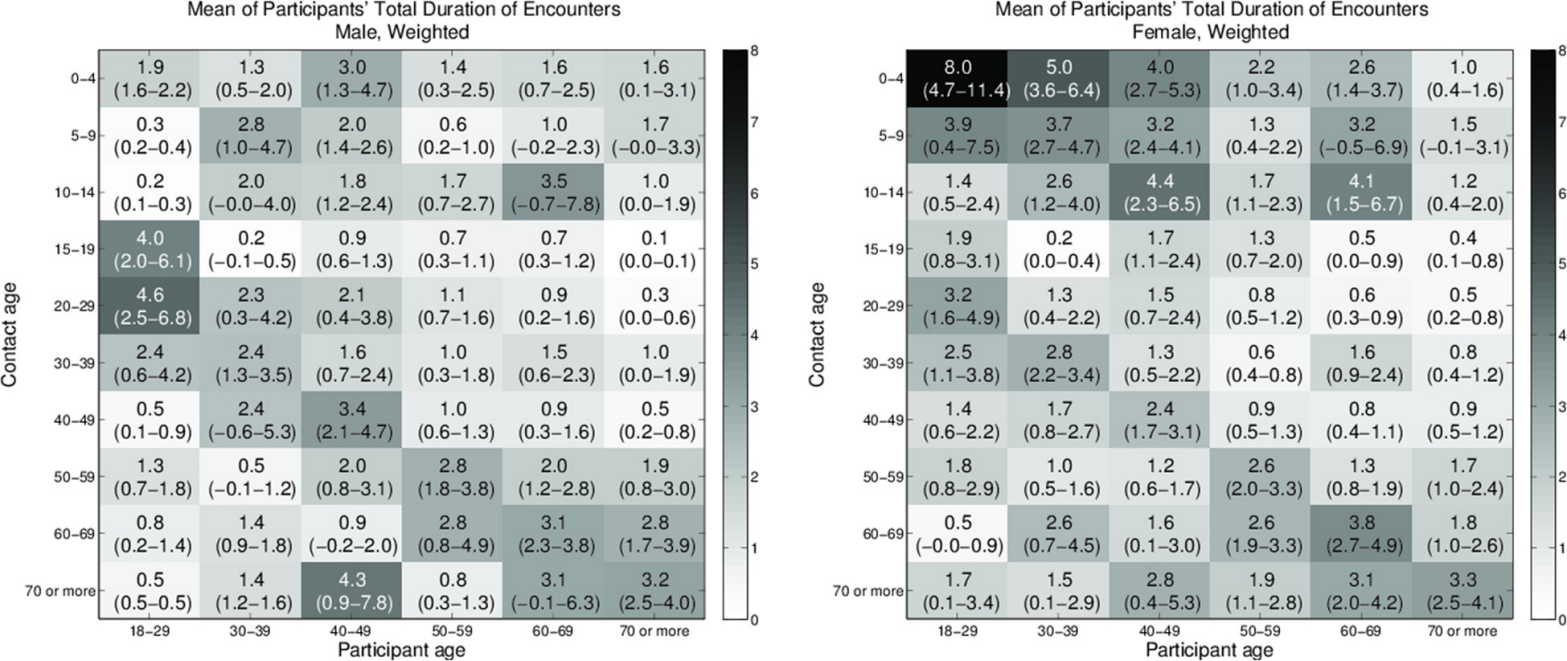
Age-based mixing matrices for participants’ total duration of encounters with listed individuals. Values are the mean of participants’ total (weighted) duration of encounters with listed individuals by age of participant and contact for male participants (*left panel*) and female participants (*right panel*). 95 % confidence intervals are shown in parentheses. Darker shading indicates larger values. While age-based assortative mixing is evident, the large duration of contact between women and pre-school age children is most notable. Raking weights are used to reduce the effects of sample bias

The duration of encounters, by age, is a further measure of mixing intensity, reported as mean hours (with 95 % confidence intervals) in Fig. 5. Again, values for men and women are reported separately. The striking difference between this figure and Fig. 4 is the dominance of interactions between women in the 18–29 years group and children of pre-school age, most likely in household settings (Fig. 5- right panel). These prolonged mixing episodes are also associated with physical contact (Additional file 4: Figure S10 (right panel)). Encounters between males aged 18–29 years, previously noted to be both frequent and close, are also prolonged (Fig. 5 (left panel), and Additional file 4: Figure S10 (left panel) for physical contact).

Heterogeneity of encounters was further assessed by 692 reports of mixing with social groups of six people or more. The median age of participants reporting group contacts was 52 years (IQR 40, 62). Median reported group size was 12 (interquartile range 9, 20), with 40 % of such contacts occurring in the workplace. Retail and hospitality, sport and recreational settings, and educational environments each accounted for approximately 10 % of listed group encounters. A large ‘other’ category included places of worship, clubs and private social gatherings. Mixing matrices for unweighted numbers of encounters and duration of encounters (in person-contact-hours) for participant contact made in a group setting are available in Additional file 4: Sections 1.10 and 1.11, where each group was categorised with one typical age (0–19 years, 20–69, 70 or more) by the participant. Given uncertain overlap between mixing groups and individually reported contacts, subsequent analyses report only on uniquely identified individuals.

### Encounters with known and unknown individuals

**Fig. 6:**
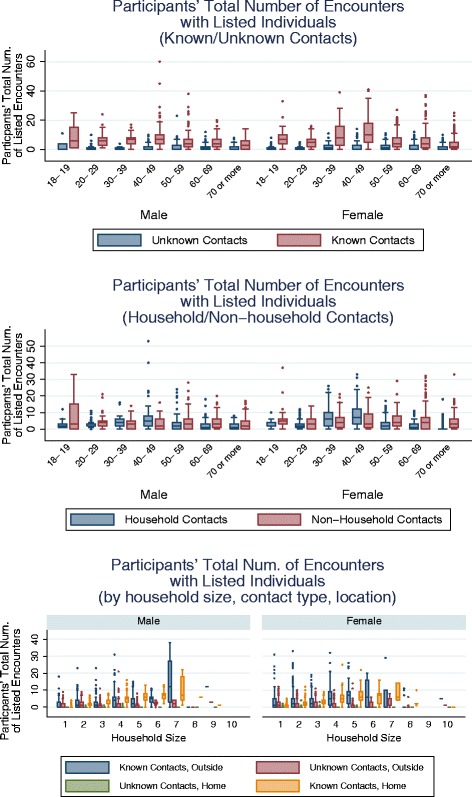
Participants’ total (weighted) number of listed encounters by type of contact individual. Boxplots for participants' total number of listed encounters by known/unknown contacts for male (*left*) and female (*right*) participants (*top panel*), household/non-household contacts for male (*left*) and female (*right*) participants (*middle panel*), and by household size, known/unknown contacts, and location of contact (Home/Outside) for male (*left*) and female (*right*) participants (*bottom panel*). Young men and women aged 30–49 years reported many more encounters with listed people than other participants. For young men, the majority are non-household members. For women, about half involved household members. In general, the number of encounters with known individuals increases with household size. Raking weights are used to reduce the effects of sample bias

Participants were asked to differentiate between encounters with individuals known to them and strangers. Young men, and women aged 30–49 years, reported many more known contacts than other respondents (Fig. 6 -left panel). In the former case, the majority of these contacts were non-household members, while among the women about half of the contacts involved family (Fig. 6 - middle). For both sexes, the total number of known contacts both inside and outside the home tended to increase with household size (Fig. 6 -bottom panel). (See Additional file 4: Figure S11 for participants’ total duration of contacts by contact type and household size.) The vast majority (75 %) of group contacts (see above) involved individuals known to participants.

### Influence of local government area of residence on encounter profiles

Summary measures of encounters, as presented in the Figures above, were compared between the LGAs surveyed in an initial exploratory analysis. While social characteristics of participants in both regions were broadly similar, some suggestions of difference emerged, warranting further evaluation. Key findings are reported in Table 1, with caution recommended in the interpretation of estimates where sample sizes were <60. Boroondara (B) residents reported mixing with more people on average in public spaces than Hume (H) residents, and similarly in retail and hospitality environments, of which more were visited [B: 1.8 (1.6, 2.0) cf H: 1.4 (1.3, 1.6), *p = 0.007*]. Hume residents tended to spend longer periods of time (hours) with others, particularly in arts and culture settings, although this latter estimate was based on a small sample size [B: 15 cf H: 9].

**Table 1:** Influence of local government area of residence on encounter profiles

	Boroondara mean	Hume mean	*P*-value
	(95 % CI)	(95 % CI)	
Number of listed encounters			
Public spaces	2.2 (1.6–2.8)	1.4 (0.9–1.8)	0.029
Retail & hospitality	3.3 (2.9–3.7)	2.7 (2.3–3.0)	0.013
Number of unique addresses			
Retail & hospitality	1.8 (1.6–2.0)	1.4 (1.3–1.6)	0.007
Participants’ total encounter duration (Hours)			
Participant total	6.2 (5.5–6.9)	7.5 (6.6–8.4)	0.028
Participants' total physical encounter duration (Hours)			
Arts & culture	0.5 (0.4–0.7)	1.1 (0.8–1.5)	0.004*
Participants' total number of known contacts			
Female 18–19	6.5 (4.6–8.4)	12.7 (6.9–18.5)	0.048*
Participants' total number of household contacts			
Female 18–19	2.4 (1.5–3.2)	5.0 (2.5–7.6)	0.05*
Participants' total number of non-household contacts			
Male, age 20–29	2.9 (1.7–4.2)	5.2 (3.7–6.8)	0.025*
Participants' total number of unknown contacts			
Female, age 50–59	2.8 (2.1–3.4)	1.5 (1.0–1.9)	0.002
Female, age 60–69	2.1 (1.5–2.7)	1.2 (0.6–1.8)	0.027
Female, age 70 or more	1.7 (1.2–2.1)	0.7 (0.3–1.0)	<0.001
Male, age 60–69	1.9 (1.2–2.6)	0.9 (0.5–1.4)	0.022

*Indicates both sample sizes are smaller than 60, which may affect validity of the *p* value

Women less than 20 years of age in Hume had contact with twice as many known individuals than in Boroondara, based on a limited number of participant reports [B: 10 cf H: 9]. Young men (20–29 years) in Hume socialised more with non-household members than those in Boroondara, although again, few participated in the study [B: 18 cf H: 19]. At the other end of the age spectrum, contact with unknown individuals was more frequent in Boroondara than Hume among females aged 50–59 and older, and males from 60 years of age.

## Discussion

Better data about patterns of social contact is needed to enable more accurate parameterisation of disease transmission models [15]. Ascertainment of conversational and physical encounters using a CATI format, while challenging to implement, yielded rich information across a large population sample. External factors known to influence mixing rates, including marked climatic variation [16], peak periods of respiratory illness [17] and school breaks [18, 19] were avoided by conducting the study over just a few months in summer and early autumn, and all in school term time. Within this context, marked heterogeneity of social behaviour was observed by location, age, gender and area of residence by attending to more detailed attributes of encounters including location, household membership and ‘strangeness’ than merely the number of contacts recorded.

Cost-efficiency was our primary motivation for trialling a CATI survey, allowing recruitment of a large and geographically well-defined population sample across two sites for this ‘proof of concept’ study. Limitations of the CATI approach included predictable biases in ascertainment [20], leading to an unrepresentative sample from each of the study areas (Additional file 1). Complexity associated with verbal recall made the interviews longer than anticipated. Participants also expressed more privacy concerns than in face-to-face interviews, resulting in incomplete or missing information in many records. In consequence, substantial time was committed to weighting, cleaning and augmenting data.

Despite these challenges, we obtained extremely detailed information on the interactions of individuals and the types of settings in which these occur. The distribution of all encounters across types of locations is similar to that reported in a variety of European countries in the POLYMOD study [2]. We did, however, note a much greater weighting of physical encounters towards the home environment than seen in that survey [2], predominantly influenced by reports of female participants. This finding should be considered in the context of related work comparing sensor and diary recordings, showing that contacts of longer duration are more likely to be recalled, and that females as a group are more accurate reporters of social encounter information [21]. It is unlikely that this difference reflects disparity in parenting behaviour between Europe and Australia, but may further relate to the nature of our diary instrument. While POLYMOD asked participants to estimate a single block of total time spent with a given individual, our respondents sequentially listed all locations and environments over the course of the day. It seems likely that this latter means of recall would allow for more accurate summation of repeat encounters, highly likely to occur in the household setting, and perhaps explaining the higher estimated proportion of household contacts.

While the overall mean number of contacts per individual was relatively low compared with a US telephone survey [20] and our own earlier work [5], this finding likely reflected the age distribution of the respondent population (Fig. 3 – right panel). When considered by age group, our observations accorded more closely with recent data from a postal and internet survey in the UK, and showed a steady decline with age [22]. While a similar decrease in sociability with age was observed in both urban and rural settings in China [23], this phenomenon is not universal. A study in rural Vietnam noted an increase in mixing rates among individuals over 40 years, persisting into older age [24]. It is not known whether such cultural differences persist after migration.

We observed far closer mixing of women with household members than men, perhaps contributing to reported differences in patterns of infection transmission. In a classic study of *Haemophilus influenzae* from the 1940s, eight times higher concordance of bacterial carriage was noted between mothers and their children than between fathers and children [25]. While times and social norms have changed substantially over the period, we recently found women to be more effective transmitters of infection than men [26], even in households where children were not present. While few studies report encounters by gender, women in a 2009 US survey reported more conversational interactions than men, although the setting in which these occurred was not stated [20]. Such disparity was not, however, observed in Vietnam suggesting that these differences cannot be universally assumed [24].

Encounter studies involving large numbers of participants have generally been targeted at whole-country level, including POLYMOD (*n* = 7290) which recruited across 8 EU countries [2], a 2010 study of almost 2000 Taiwanese residents [27] and a recently published UK-wide survey of more than 5000 respondents [22]. In contrast, a North Carolina telephone survey of social encounters recruited almost 4000 participants from four pre-specified counties, but no specific rationale was given for their selection [20]. A large Chinese study recruited 1821 individuals across a geographical zone spanning urban and rural environments, to consider the influences of distance and population density on social interactions [23]. Our explicit strategy was to densely sample two diverse areas of one major city in Australia, to understand qualitatively reported small area socio-demographic and environmental influences on social network heterogeneity [7]. The preliminary analyses presented here suggest varying location preferences, and gendered differences in mixing beyond the household unit, by area of residence, that will be explored in more detail in subsequent work. In particular, the geographical extent of social networks, and the overarching influences of both household and area level advantage, are of interest.

## Conclusions

Mixing matrices based on physical encounter data such as these have been incorporated in age-structured transmission models, and validated through their ability to reproduce age-dependent infection profiles in cross-sectional population serosurveys [28]. More recently, encounter measures have also been correlated with laboratory confirmed evidence of respiratory infection at the individual level [29, 30]. Assumptions regarding the number, duration and clustering of contacts in model frameworks all have significant implications for the simulated spread of infection [31], and optimal strategies for its control [3]. Our hypothesis is that differences in social behaviour may contribute, at least in part, to the increasing infection risk observed with disadvantage [32], mediated at the level of the household, neighbourhood or workplace [33].

The incorporation of geographical space into representations of social networks is a recognised challenge in the field of infectious disease modelling [34]. As diversity increases in modern cities [35], location of residence encodes a far greater variety of attributes that may influence social behaviour than merely distance between individuals, or proximity to mixing locations. Spatial differences in culture, advantage and environment will likely exert pronounced effects on network characteristics, with local implications for infectious disease transmission and risk. Further evaluation of this dataset will involve detailed profiling of small areas, to seek additional evidence of both household and neighbourhood level influences on social behaviour and development of network models of infection spread, to investigate the likely implications of difference for disease.

## Additional files

Additional file 1:
**Cleaning, Geocoding and Weighting.** Additional details on data cleaning, geocoding of addresses, sample bias and sample weighting using raking. (PDF 900 kb) Additional file 2:
**Questionnaire.** Additional file descriptions text (including details of how to view the file, if it is in a non-standard format). (PDF 130 kb) Additional file 3:
**Contact Diary.** Additional file descriptions text (including details of how to view the file, if it is in a non-standard format). (PDF 61 kb) Additional file 4:
**Additional Results.** Additional results including details on number and duration of physical contact encounters, and duration of encounters for each household size by contact type (known/unknown) and location type (home/outside). (PDF 1275 kb)
