# Supplementary material for: Social encounter profiles of greater Melbourne residents, by location – a telephone survey
Source: BMC Infect Dis. 2015 Nov 2;15:494. doi: 10.1186/s12879-015-1237-9 (PMC4631075; doi:10.1186/s12879-015-1237-9)
Supplement: Additional file 1: — Cleaning, Geocoding and Weighting. Additional details on data cleaning, geocoding of addresses, sample bias and sample weighting using raking. (PDF 900 kb) [file 12879_2015_1237_MOESM1_ESM.pdf]

Additional file 1: Cleaning, Geocoding and Weighting

Contents

1 Introduction 2
2 Address Accuracy and Cleaning 2
2.1 Sources 2
2.2 Address Linking 3
2.3 Cleaning Summary 3
3 Time Consistency and Cleaning 4
4 Geocoding 5
4.1 Bing Map Queries and Manual Geocoding 5
4.2 Post-gecoding confirmation 5
5 Biased Sampling, Raking and Sampling Weights 6
5.1 Data 6
5.1.1 Age groups 6
5.1.2 Gender 7
5.1.3 Marital Status 7
5.1.4 Australia-Born 8
5.1.5 Household size 8
5.1.6 Household Types 8
5.1.7 Home Ownership 9
5.2 Weighting Method 9
5.3 Goodness of Fit Results after Weighting 10
6 Tables Demonstrating Bias in CATI Data 12
6.1 Selected Medians and Averages 12
6.2 Age of participants (census data for age 18+) 12
6.3 Gender (census data for age 18+) 13
6.4 Age/Gender (census data for age 18+) 13
6.5 Australia Born (age 18+) 14
6.6 Top 5 countries of birth of participants (age 18+) 14
6.7 Speaks Only English at Home (age 18+) 15
6.8 Registered Marital Status (age 18+) 15
6.9 Achieved Year 12* (age 18+) 16
6.10 Same Address as 1/5 years ago (age 18+) 16
6.11 Household size 17
6.12 Household Income 17
6.13 Home Ownership 18
7 References 19

1 Introduction

This document describes the cleaning, geocoding and weighting of data collected using computer-assisted telephone interviewing (CATI) in two cities (Boroondara and Hume) surrounding Melbourne, Australia in 2013. Participants were asked to provide demographic and socio-economic details on themselves and a partner (if any). Importantly, for the day preceding the interview, they were also asked to give details on every location they visited, when they left that location, whom they had contact with, and how long that contact lasted.

The nature of the CATI style of data collection is that an interviewer records responses heard over the phone. Compared to a participant recording responses directly, this introduces additional scope for errors (e.g., phonetic spelling errors of street names.) The rapid pace of an interview can mean typographic or other errors are introduced and common business names are recorded instead of addresses. A range of interviewers and participants can mean multiple ways are used to describe the same place (not an error, but still a problem for using the data for research.)

An additional problem common to most surveys is bias such that the sample does not closely re-semble the target population in various ways (e.g., demography and socio-economic indicators). Weighting of survey samples to reduce bias is a common approach to address this problem. Of the available weighting techniques, iterative proportional fitting (i.e., raking) has become a common used technique due to its flexibility where marginal census totals, but not joint distributions, are available for variables of interest.

In this document we describe the data cleaning, geocoding and weighting that was performed.

2 Address Accuracy and Cleaning

2.1 Sources

All suburbs were corrected to standard Australia Post names [1]. Where maps were consulted, Mel-ways [2] based on Melways Edition 38, and occasionally the Google Maps website [3] were used. The Melways online site was used to decide if a street name uniquely identified the location suburb.

Corporate website locators were used to standardize addresses for big-box and other chain stores. "Famous Landmark, Address Not Required" in the street address field was resolved by using other recorded information to identify common locations, or the sequence of locations together with times to narrow down possible missing location suburb. (i.e., for times within 5-10 minutes within a known location).

For missing postcodes with references to Neighbour, or Neighbourhood starting or ending near home, addresses are assumed to be in the same postcode.

2.2 Address Linking

Address linking is the process of connecting a contact location to an address, which is a many-to-one relationship. The following rules were used to create "keys" with the details shown that were the basis for identifying addresses common to contact locations.

If the postcode was available:

- for houses: participant ID, street address, suburb, postcode,
- for other locations: street address, suburb, postcode.

If the postcode was not available:

- for transport location types: location id assigned by the interviewer
- for others: location id assigned by the interviewer

with the proviso that if a location was revisited by the same participant (e.g., using supplementary information like the location description) those locations were considered to be the same address.

People were assumed to have one home address in the study area. In the case of multiple contact locations of "home" location type, ones with incomplete or refused addresses were still assumed to be the same. In the case where no address details were provided, the postcode used for screening participants who lived in the study area was used to indicate where the participant lived. Six participants never visited their home.

2.3 Cleaning Summary

	**Before**	**After**	**Change**
Number of LOCID in source data	8786	8787	1
Number of non-blank street addresses	4981	5489	508
Number of non-blank location suburbs	4665	5825	1160
Number of suburbs changed (including effect of spaces)			3905
Number of suburbs changed (ignore extra spaces)			1461
Number of non-blank suburbs changed (ignore extra space)			307
Number of addresses with spelling corrected			>133
Number of addresses intervened somehow			>1950

Table 1 Cleaning Summary

3 Time Consistency and Cleaning

Three main problems with recorded times were corrected in the cleaning phase. The first of these were AM/PM errors (e.g., a participant is recorded to leave locations 1, 2, 3 at 10:30 AM, 12:15 *AM*, 1:00 PM). The second time clearly should be PM.

The second common problem with recorded times was an inconsistency between a participant's time at a location and their time in contact with a person at that location. Obviously a basic rule must apply where someone's contact duration with a person at a location is not more than their duration at their location. Due to how the interviews were structured, a participant's duration at a location is more reliable. Thus we used the following method to resolve the inconsistency, and allow for possible rounding of responses:

- If a participant's duration at a location is zero, cap the contact duration with a person at 5 minutes (to allow for possible rounding).
- If a participant's duration at a location is greater than zero, cap the contact duration with a person at that location at the location duration.

The third common problem arose from an oversight in the survey design. The approach of the survey was to ask when the participant left each location. From this can be inferred their arrival time at each location, and thus duration at each location. But, it is also helpful to know when the participant woke up in the morning, since that marks the beginning of possible activities or contact with others. Similarly, at the end of the day, when they went to sleep marks the end of possible activities or contact. Since these times were not collected, two rules were created for the study.

- Morning start rule:
  If a respondent starts their day at home, assume a start time of 7 AM, unless they report leaving that location before 7 AM, in which case assume a start time of one hour before the reported time leaving the home.
- Night ending rule:
  If a respondent ends their day at home, assume an end time of 11 PM, unless they report arriving at that location after 11 PM, in which case assume an end time of one hour after the reported arrival time at home.

4 Geocoding

Geocoding is the process of assigned latitude/longitude coordinates to addresses. CATI addresses were geocoded using a mix of API queries (mainly Bing Maps [4], but also MapQuest [5] and OpenStreetMaps [6]) and manual location finding via the Google Maps website [3]. Because of Google Maps terms of use, geocode results from that API queries cannot be saved.

In the process of geocoding, additional address cleaning was necessary. Typical reasons were spelling mistakes in street names, alternate street names, or an incorrect suburb provided. This phase of address cleaning was particularly time-intensive.

4.1 Bing Map Queries and Manual Geocoding

The majority of geocoding was performed by issuing queries to APIs. To develop confidence in those results a number of steps were taken. Results from Bing Maps/Mapquest/OpenStreetMaps were compared with that from Google Maps. Automatic rules and manual checking was performed based on the "quality" returned in the query result and the distance between the Bing Maps/Mapquest/OpenStreetMaps point and the Google Maps point. Some flexibility is required in declaring a match because Google Maps assigns geocodes to addresses and points of interest in the interior of a building's lot, not at the street. Geocodes for large locations (e.g. shopping malls, big box stores) may differ considerably on account of this difference.

A number of automatic rules for matching non-Google and Google geocodes were used.
1) The address string is blank (an empty address).
2) The Bing Maps geocode passes basic checks on match quality and latitude and:
   i. the geocode is within 50 m of the Google Maps point for an address that starts with a number, or
   ii. the geocode is a street intersection and the Bing Maps returned quality says the EntityType is RoadIntersection, or
   iii. the geocode is within 100m of the Google Maps point for a street without a street number.
3) The street field is blank, the returned address postcode is correct, and the quality returned by Bing Maps says the EntityType is a postcode.

Basic checks in this context include geocodes within a rectangle containing the state of Victoria (for Victorian addresses) or the country of Australia (for other Australian addresses). A number of geocodes that did not automatically match were manually confirmed by placing the geocode onto a map. Bing Maps results for cities outside Victoria, Australia (esp. without streets) were allowed to differ much more from Google results. Additional geocoding was performed manually using the Bing Maps and/or Google Maps website to obtain a correct geocode for each address, as best as possible.

4.2 Post-gecoding confirmation

The straight-line distance between all geocodes was calculated. This identified some cases where different address records were really for the same place. All pairs of geocodes within 80 m were checked and confirmed to be distinct. (This does not apply to suburb-only addresses, which are a catch-all when street name is unknown.)

5 Biased Sampling, Raking and Sampling Weights

5.1 Data

The CATI survey is a stratified sample where the strata are the two communities sampled: Boroondara (n=650) and Hume (n=657). As is common for sample surveys, the results are biased relative to the 2011 Australian census totals for those communities in the following ways. (Section 6 provides detailed tables demonstrating how the CATI sample differs from the 2011 Australian census totals.)

- The fraction of the sample over 50 is too high.
- The fraction of the sample that is female is too high.
- There are too many small household.
- There are too many Australia-born participants.
- There are too many English only households.
- There are too many “Widowed” participants and not enough “never married” participants.
- There are too many participants that achieved year 12 (i.e., completed high school).
- There are too many participants living at the same address as one and five years ago.
- Too many participants own their house outright and not nearly enough are renting.

In addition many participants didn’t report household income (175 refused, 218 don’t know), and the underreporting is possibly biased such than lower incomes are more likely to be reported. To reduce these biases, raking was used to generate post-sampling weights, sometimes called pweights. This section describes the data that was used and gives corresponding Australia Bureau of Statistics (ABS) items from the 2011 census. Census totals for ABS items are available using the ABS Table-Builder tool [7].

5.1.1 Age groups

Age related bias was a key problem to be addressed by weighting. Note that because participant age is reported far less than the categorical “age group” in the survey, age categories derivable from the “age group” used in the data collection are preferable. Some combining of age categories was helpful:

- Age group 18-19, is small and doesn’t correspond to a census age group. It is helpful to combine with age group 20-29.
- Age groups 20-29 and 30-39 are really under sampled (e.g. in Boroondara Male 30-39: 2.4% in sample vs. 15.1% in census). To maintain some age diversity it is helpful to combine 30-39 with 40-49, instead of 18-29.

The result was five age categories as shown in the table.

CATI 7 category	5 category for weighting
18- 19	18- 29
20- 29	18- 29
30- 39	30- 49
40- 49	30- 49
50- 59	50- 59
60- 69	60- 69
70 or more	70+ years
Refused	Refused

Table 2 Recoded Age Categories for Weighting

5.1.2 Gender

Gender was also included to address gender related bias. Because of the quality of the survey and census data available, the joint distribution of age (five categories) and gender (two categories) was used.

5.1.3 Marital Status

As household structure is expected to be a key use of the data, bias in the marital status of participants was addressed. This data was obtained in the survey via the question:
“Which of the following best describes your current marital status?” for which the possible responses were

1. Married
2. Living with a partner
3. Widowed
4. Divorced
5. Separated, or
6. Never married
7. (Don’t know)
8. (Refused)

This presents a problem when comparing with census totals (registered marital status; MSTP) because that item does not include an option “living with partner” and Australia does not currently recognize same-sex marriage. An alternative item from the census (social marital status; MDCP) is not exactly analogous (*not* married vs. *never* married). Without a census category to compare with “living with partner”, those participants would be assigned weight zero and excluded from all weighted analyses. As an alternative, the joint counts of MSTP and MDCP were used to create census totals analogous to the categories used in the CATI survey in the manner shown in the table below.

		MDCP			
		Married in a registered marriage	Married in a de facto marriage	Not married	Not applicable
MSTP	Married	Married	Live w Partner	Married	Married
	Widowed	Widowed	Live w Partner	Widowed	Widowed
	Divorced	Divorced	Live w Partner	Divorced	Divorced
	Separated	Separated	Live w Partner	Separated	Separated
	Never married	Never married	Live w Partner	Never married	Never married
	Not applicable	exclude	exclude	exclude	exclude

Table 3 Allocation of MSTP/MDCP Census Numbers to CATI Categories

Because of the low number of occurrences of some categories in the sample, further combining of categories was necessary. The result was three categories: “coupled”, “never married”, and “other” as shown in the table below. Weights were generated using this three category system, but goodness-of-fit is reported using both the five categories of the census registered marital status, and the six category system of the CATI survey that includes “living with partner”, and as imputed for the census totals.

CATI	Census: Registered Marital Status	Census: Social Marital Status	New Weighting Category
Married	Married	Married in a regis-tered marriage	Coupled
Living with a partner	?	Married in a de facto marriage	Coupled
Widowed	Widowed		Other
Divorced	Divorced		Other
Separated	Separated		Other
Never married	Never married	Not married	Never Married
Don't Know	Not Applicable	Not applicable	

Table 4 Marital Status Coding Schemes

5.1.4 Australia-Born

To address bias related to the country of origin of the participants, a binary variable indicating whether a participant was born in Australia or not, was derived and used. This was compared with totals derived from 2011 census results (country of birth of person; BPLP). This was preferable to weighting on individual countries because there are so few (or no) participants sampled from some.

5.1.5 Household size

As household structure is expected to be a key feature of research informed by this data, bias in the household size was addressed. The household size of a participant was derived from the number of household contacts reported for the household, and includes the participant. This was compared to totals from the 2011 census (household composition by number of persons usually resident; NPRD). For the purposes of weighting, household sizes were collapsed into three categories: 1, {2,3}, and 4 or more.

5.1.6 Household Types

A notion of "household type" was found to be helpful to achieve acceptable goodness-of-fit for marital status and household size simultaneously. Categories used for household types are taken from an analogous ABS category (family household composition; HCFMD) counting household type by household (not by person). (The use of random digit dialling of landlines approximately implies by household.) The table below shows the categories that were used, and how several smaller categories were collapsed. Household type for the CATI data was derived from details of the household links which indicate, in part, other relatives living in the household.

Census HCFMD code	Household type
**11**	One family household: Couple family with no children
**12**	One family household: Couple family with children
**13**	One family household: One parent family
**14**	One family household: Other family
**31**	Lone person household
	Other classifiable household<br>(incl. 21-28, 32; not 33, 34 , N/A)

Table 5 Census HCFMD Household Types used for Weighting

5.1.7 Home Ownership

To address bias in home ownership this was also included in the raking scheme. Population data for home ownership by household is available in Table B32 (tenure type, TTEN) of the Basic Community Profile for Boroondara [8] and Hume [9]. The seven category system of the CATI survey was re-coded into the four major four categories (Owned outright, Owned with a mortgage, Renting, and Other) reported for the census as shown in the table. A fifth category "Missing" was also used, and is described below. Note that of the six types of data used for weighting, home ownership is probably the one most closely connected with "financial" resources.

Census Home Ownership Types	CATI Home Ownership Types
**Owned outright**	Owned outright
**Owned with a mortgage**	Owned with a mortgage
**Owned with a mortgage**	Being purchased under a rent/buy scheme
**Renting**	Being rented privately
**Renting**	Public housing
**Other**	Being occupied rent free
**Other**	Other(Specify)

Table 6 Home Ownership Category Scheme

5.2 Weighting Method

Raking was performed in Stata [10] using the survwgt add-on program [11] (available in Stata by typing "ssc install survwgt"). The weights were obtained using the following data:

1. the joint distribution of five age categories and gender (5 x 2 categories)
2. marital status (3 categories)
3. Australia-born (yes/no)
4. household type (six categories)
5. household size (1,2,3,4,5,6+)
6. home ownership (4 categories + Missing)

For these data, there is some missing data in Boroondara (age category n=0; marital status n=9) and Boroondara (age category n=2; marital status n=7). Participants with missing responses in these data would result in zero weights and so be excluded from future analysis. One age category response was derivable from other data (age 70+). The remaining age category and the 16 marital status responses were imputed. The marital status responses were imputed using a multinomial logit model using five predictors (age x gender, marital status, Australia-born, household type and household size) and pweights obtained from those responses without missing values.

To address more numerous non-response in the home ownership data (Boroondara: n=30; Hume: n=21) a re-allocation approach was used [12]. Non-response records were allocated to a fifth "Missing" category. Control totals for the "Missing" category were set to 30 (Boroondara) and 21 (Hume). Control totals for the other four categories were reduced by a corresponding fraction (Boroondara: 620/650; Hume: 636/657).

As is common with the raking technique, extreme weights were truncated (to produce smaller weights). After truncation the weights were renormalised to maintain the original sum (i.e., the sample size) for Boroondara and for Hume. Reducing the weights is recognised to reduce variance at the expense of increasing bias. Weights were truncated at 7 (Boroondara) and 6 (Hume) which were found to be the smallest values for which goodness-of-fit tests of raked variables against corre-

sponding census totals did not reject at the 5% level. (In the case of age and marital status, both original CATI and recoded categories were tested against their appropriate census totals.) The total weight for records with imputed age and/or marital status is 13.9 (Boroondara) and 7.2 (Hume).

5.3 Goodness of Fit Results after Weighting

The table below shows goodness-of-fit results (as P-values unless otherwise stated) between various CATI data and corresponding census totals. The characteristics tested reflect key features of demography or socio-economic status often considered relevant in survey sample weighting, or household features expected to be important in anticipated analyses. Adjusted Wald tests of significance were performed to test for the equality of the mean between sample and census (for binary responses), and to test whether the data source (sample vs. census) was significant for unordered categorical and ordered categorical data.

It is clear that prior to weighting the sample is biased in many ways. Following weighting all predictors used for weighting fit to an acceptable standard and most others fit well too. In particular, the weighted sample captures key demographic distributions of age, gender, marital status and the proportion born in Australia. The weighted sample also captures key socio-economic indicators of household income and home ownership. The latter result is particularly striking since home ownership is not explicitly using in the weighting. The weighted sample also captures key features of the household size including the average household size and the distribution of household sizes.

		Sample (no weights)		Raking weights	
	weighting:	none	none	{age (5 categories) x gender},<br>marital status (Coupled, Never married, Other),<br>Aus-born,<br>household type, household size,<br>home ownership	
**Test type**	**Characteristic \ P-values**	Boroondara	Hume	Boroondara	Hume
O	Age (age 18+, 7 categories)	<1e-4	<1e-4	0.06	0.22
B	Gender (age 18+)	<1e-4	<1e-4	0.90	0.98
B	Australia born (age 18+)	<1e-4	<1e-4	0.52	0.90
B	Speaks Only English at Home (age 18+)	<1e-4	<1e-4	0.24	0.001
U	Registered Marital status (age 18+) (excludes “living w partner”)	<1e-4	<1e-4	0.52	0.11
U	CATI Current Marital status (age 18+) (allocated census values)	not evaluated		0.73	0.85
O	Household size (1,2,3,4,5,6+)	<1e-4	0.007	0.43	0.67
	Average household size (B: 2.6, H: 3.1)	2.40	3.00	2.7	3.1
O	Average household size 95% CI:	2.3-2.5	2.9-3.1	2.5-2.9	2.9-3.3
O	Household income	0.001	0.0003	0.77	0.76
U	Home ownership	<1e-4	<1e-4	0.43	0.74

Table 7 Goodness-of-fit Results Before and After Weighting

Notes
yellow: rejects at 5% significance; green: does not reject

Test type
Binary (B): adjusted Wald test after computing mean (which obtains the proportion for 0/1 values)
Ordered categorical (O): adjusted Wald test after ordered logistic regression
Unordered categorical (U): adjusted Wald test after multinomial logistic regression

6 Tables Demonstrating Bias in CATI Data

The following tables compare key demographic, household and socio-economic indicators between the CATI data and the 2011 Australian census for both Boroondara and Hume. Proportions that differ by more than 5% are highlighted, as this is sometimes cited as the threshold beyond which bias should be addressed.

6.1 Selected Medians and Averages

	Boroondara LGA		Hume LGA	
	CATI	Census	CATI	Census
Median age	62	38	54	33
Median household income (weekly)	1300-1599 (but 6 away from the next category)	1893	1000-1299	1214
Average household size (assumes mean)	2.4	2.6	3.0	3.1

Table 8 Comparison of Selected Statistics Between Sample and 2011 Census for Both Cities

6.2 Age of participants (census data for age 18+)

Age	Count	Boroondara Sample Proportion	Boroondara LGA Proportion	Count	Hume Sample Proportion	Hume LGA Proportion
18- 19	15	0.023	0.039	23	0.035	0.043
20- 29	34	0.052	0.195	53	0.081	0.204
30- 39	21	0.032	0.150	69	0.105	0.198
40- 49	81	0.125	0.190	112	0.171	0.207
50- 59	135	0.208	0.168	158	0.241	0.160
60- 69	159	0.245	0.120	131	0.200	0.107
70 or more	205	0.315	0.138	109	0.166	0.081
Refused	0			2		
**Total**	**650**	**1.000**	**1**	**657**	**1.000**	**1**

Table 9 Comparison of Age Distribution between Sample and 2011 Census Sample (census) cells are shown in yellow when they are at least 5% larger than the corresponding census (sample) value.

6.3 Gender (census data for age 18+)

Row Labels	Count	Boroondara Sample Proportion	Boroondara LGA Proportion	Count	Hume Sample Proportion	Hume LGA Proportion
Female	398	0.61	0.53	395	0.60	0.51
Male	252	0.39	0.47	262	0.40	0.49
**Grand Total**	**650**	**1.000**	**1.000**	**657**	**1.000**	**1.00**

Table 10 Comparison of Gender Distribution between Sample and 2011 Census Sample (census) cells are shown in yellow when they are at least 5% larger than the corresponding census (sample) value.

6.4 Age/Gender (census data for age 18+)

Male Age	Count	Boroondara Sample Proportion	Boroondara LGA Proportion	Count	Hume Sample Proportion	Hume LGA Proportion
18- 19	5	0.020	0.042	14	0.054	0.044
20- 29	18	0.071	0.211	19	0.073	0.207
30- 39	6	0.024	0.151	17	0.065	0.198
40- 49	33	0.131	0.186	46	0.176	0.207
50- 59	50	0.198	0.170	62	0.238	0.161
60- 69	60	0.238	0.123	63	0.241	0.107
70 or more	80	0.317	0.117	40	0.153	0.076
Refused				1		
**Total**	**252**	**1.000**	**1.000**	**262**	**1.000**	**1.000**

Female Age	Count	Boroondara Sample Proportion	Boroondara LGA Proportion	Count	Hume Sample Proportion	Hume LGA Proportion
18- 19	10	0.025	0.036	9	0.023	0.041
20- 29	16	0.040	0.181	34	0.086	0.201
30- 39	15	0.038	0.149	52	0.132	0.198
40- 49	48	0.121	0.193	66	0.168	0.208
50- 59	85	0.214	0.166	96	0.244	0.159
60- 69	99	0.249	0.118	68	0.173	0.106
70 or more	125	0.314	0.157	69	0.175	0.086
Refused				1		
**Total**	**398**	**1.000**	**1.000**	**395**	**1.000**	**1.000**

Table 11 Comparison of Age Distribution by Gender between Sample and 2011 Census Sample (census) cells are shown in yellow when they are at least 5% larger than the corresponding census (sample) value.

6.5 Australia Born (age 18+)

	Count	Boroondara Sample Proportion	Boroondara LGA Proportion	Count	Hume Sample Proportion	Hume LGA Proportion
Australia	497	0.76	0.66	446	0.68	0.57
non-Australia	153	0.24	0.34	211	0.32	0.43
**Total**	**650**	**1**	**1**	**657**	**1**	**1**

Table 12 Comparison of Proportion of Participants Australia-born between Sample and 2011 Census Sample (census) cells are shown in yellow when they are at least 5% larger than the corresponding census (sample) value.

6.6 Top 5 countries of birth of participants (age 18+)

	Count	Boroondara Sample Proportion	Boroondara LGA Rank	Boroondara LGA Proportion
Australia	497	0.76	1	0.66
United King-dom	42	0.06	3	0.05
New Zealand	18	0.03	6	0.02
Malaysia	13	0.02	5	0.02
India	8	0.01	4	0.03
**Grand Total**	**650**	**0.89**		**0.77**

Table 13 Comparison of Sample and 2011 Census Proportion Born from CATI Top Five Countries of Birth (Boroondara) Sample (census) cells are shown in yellow when they are at least 5% larger than the corresponding census (sample) value. In the 2011 census the top five countries of birth are Australia, China (5.0%), the UK, India, and Malaysia. Note that in the CATI sample China ranks sixth with eight people.

	Count	Hume Sample Proportion	Hume LGA Rank	Hume LGA Proportion
Australia	446	0.68	1	0.57
United King-dom	43	0.07	5	0.03
Italy	22	0.03	6	0.03
Turkey	14	0.02	3	0.05
India	12	0.02	4	0.03
**Grand Total**	**657**	**0.82**		**0.72**

Table 14 Comparison of Sample and 2011 Census Proportion Born from CATI Top Five Countries of Birth (Hume) Sample (census) cells are shown in yellow when they are at least 5% larger than the corresponding census (sample) value. In the 2011 census the top five countries of birth are Australia, Iraq (5.5%), Turkey, India, and the UK. Note that in the sample Iraq is tenth with seven people.

6.7 Speaks Only English at Home (age 18+)

	Count	Boroondara Sample Proportion	Boroondara LGA Proportion	Count	Hume Sample Proportion	Hume LGA Proportion
English Only	543	0.835	0.743	480	0.731	0.563
No	107	0.165	0.257	177	0.269	0.437
**Total**	**650**	**1.000**	**1.000**	**657**	**1.000**	**1.000**

Table 15 Comparison between Sample and 2011 Census of Proportion of English-only Households Sample (census) cells are shown in yellow when they are at least 5% larger than the corresponding census (sample) value.

6.8 Registered Marital Status (age 18+)

Marital Status	Count	Boroondara Sample Proportion	Boroondara LGA Proportion	Count	Hume Sample Proportion	Hume LGA Proportion
Married	341	0.551	0.533	379	0.613	0.553
Widowed	99	0.160	0.056	63	0.102	0.044
Divorced	50	0.081	0.059	55	0.089	0.074
Separated	15	0.024	0.018	19	0.031	0.039
Never married	114	0.184	0.334	102	0.165	0.290
**Total**	**619**	**1.000**	**1.000**	**618**	**1.000**	**1.000**
Living with partner	22			32		
Refused	8			4		
Don't know	1			3		
**Grand Total**	**650**			**657**		

Table 16 Comparison between Sample and 2011 Census of Martial Status Note that the CATI category “Living with Partner” does not correspond to a 2011 census registered marital status. Sample (census) cells are shown in yellow when they are at least 5% larger than the corresponding census (sample) value. Non-response is omitted from the comparison as shown.

6.9 Achieved Year 12* (age 18+)

	Count	Boroondara Sample Proportion	Boroondara LGA Proportion	Count	Hume Sample Proportion	Hume LGA Proportion
Yes	575	0.886	0.806	443	0.680	0.501
No	74	0.114	0.194	208	0.320	0.499
**Total**	**649**	**1.000**	**1.000**	**651**	**1.000**	**1.000**
Refused	0			1		
Don't know	0			3		
Other (Specify)	1			2		
**Grand Total**	**650**			**657**		

Table 17 Comparison between Sample and 2011 Census of Proportion of Participants who Achieved Year 12* Sample (census) cells are shown in yellow when they are at least 5% larger than the corresponding census (sample) value. Non-response is omitted from the comparison as shown.

*Note: "achieved year 12" in Australia may be known as "completed high school" in other jurisdictions.

6.10 Same Address as 1/5 years ago (age 18+)

	Count	Boroondara Sample Proportion	Boroondara LGA Proportion	Count	Hume Sample Proportion	Hume LGA Proportion
Yes	648	0.998	0.845	649	0.989	0.882
No	1	0.002	0.155	7	0.011	0.118
**Total**	**649**	**1.000**	**1.000**	**656**	**1.000**	**1.000**
Other/Not stated	1			1		
**Grand Total**	**650**			**657**		

Table 18 Comparison between Sample and 2011 Census of Proportion of Participants Living at Same Address (One Year) Sample (census) cells are shown in yellow when they are at least 5% larger than the corresponding census (sample) value. Non-response is omitted from the comparison as shown.

	Count	Boroondara Sample Proportion	Boroondara LGA Proportion	Count	Hume Sample Proportion	Hume LGA Proportion
Yes	601	0.926	0.602	568	0.866	0.647
No	48	0.074	0.398	88	0.134	0.353
**Total**	**649**	**1.000**	**1.000**	**656**	**1.000**	**1.000**
Other/Not stated	1			1		
**Grand Total**	**650**			**657**		

Table 19 Comparison between Sample and 2011 Census of Proportion of Participants Living at Same Address (Five Years) Sample (census) cells are shown in yellow when they are at least 5% larger than the corresponding census (sample) value. Non-response is omitted from the comparison as shown.

6.11 Household size

Household size	Count	Boroondara Sample proportion	Boroondara LGA Proportion	Count	Hume Sample Proportion	Hume LGA Proportion
1	185	0.285	0.242	92	0.140	0.163
2	240	0.369	0.310	213	0.324	0.267
3	74	0.114	0.158	114	0.174	0.187
4	97	0.149	0.188	143	0.218	0.210
5	45	0.069	0.080	65	0.099	0.105
6+	9	0.014	0.022	30	0.046	0.068
**Grand Total**	**650**	**1.000**	**1.000**	**657**	**1.000**	**1.000**

Table 20 Comparison between Sample and 2011 Census of Participants' Household Size Household size is the reported number of people living within the participant's home. Sample (census) cells are shown in yellow when they are at least 5% larger than the corresponding census (sample) value.

	Boroondara CATI	Boroondara LGA Census	Hume CATI	Hume LGA Census
**Average Household size**	2.4	2.6	3	3.1

Table 21 Comparison between Sample and 2011 Census of Average Household Size

6.12 Household Income

	Count	Boroondara Sample Proportion	Boroondara LGA Proportion	Count	Hume Sample Proportion	Hume LGA Proportion
Nil income	3	0.007	0.021	1	0.002	0.014
$1-$399 per week	30	0.068	0.079	62	0.131	0.101
$400-$599 per week	37	0.084	0.064	49	0.104	0.102
$600-$799 per week	28	0.063	0.058	45	0.095	0.101
$800-$999 per week	36	0.082	0.062	57	0.121	0.092
$1000-$1999 per week	131	0.297	0.239	178	0.376	0.344
$2,000 or more per week	176	0.399	0.477	81	0.171	0.246
**Total**	**441**	**1.000**	**1.000**	**473**	**1.000**	**1.000**
Refused	101			74		
Don't know	108			110		
**Grand Total**	**650**			**657**		

Table 22 Comparison between Sample and 2011 Census of Distribution of Household Income Sample (census) cells are shown in yellow when they are at least 5% larger than the corresponding census (sample) value. Non-response is omitted from the comparison as shown.

6.13 Home Ownership

	Count	Boroondara Sample Proportion	Boroondara LGA Proportion	Count	Hume Sample Proportion	Hume LGA Proportion
Owned outright	424	0.684	0.410	319	0.502	0.297
Owned with a mortgage	144	0.232	0.297	257	0.404	0.487
Rented (incl. public housing)	46	0.074	0.287	51	0.080	0.210
Other (Specify)	6	0.010	0.006	9	0.014	0.006
**Total**	**620**	**1.000**	**1.000**	**636**	**1.000**	**1.000**
Refused/Not stated	30			21		
**Grand Total**	**650**			**657**		

Table 23 Comparison between Sample and 2011 Census of Distribution of Home Ownership Sample (census) cells are shown in yellow when they are at least 5% larger than the corresponding census (sample) value. Non-response is omitted from the comparison as shown.

7 References

1. **Postcode Search** [http://auspost.com.au/apps/postcode.html]
2. **Online Maps** [http://www.melway.com.au/online-maps/]
3. **Google Maps** [http://maps.google.com.au/maps]
4. **Locations API** [http://msdn.microsoft.com/en-us/library/ff701715.aspx]
5. **MapQuest Platform Web Services** [http://www.mapquestapi.com/]
6. **Nominatim - OpenStreetMap Wiki** [http://wiki.openstreetmap.org/wiki/Nominatim]
7. **TableBuilder** [http://www.abs.gov.au/websitedbs/censushome.nsf/home/tablebuilder]
8. Australian Bureau of Statistics: *2011 Census of Population and Housing: Basic Community Profile (Boroondara).* 2012.
9. Australian Bureau of Statistics: *2011 Census of Population and Housing: Basic Community Profile (Hume).* 2012.
10. StataCorp: *Stata Statistical Software: Release 10.* College Station, TX: StataCorp LP; 2007.
11. Winter N: *'SURVWGT': module to create and manipulate survey weights.*
12. Battaglia MP, Hoaglin DC, Frankel MR: **Practical Considerations in Raking Survey Data.** *Survey Practice* 2013, **2**.
