# Supplementary material for: Social encounter profiles of greater Melbourne residents, by location – a telephone survey
Source: BMC Infect Dis. 2015 Nov 2;15:494. doi: 10.1186/s12879-015-1237-9 (PMC4631075; doi:10.1186/s12879-015-1237-9)
Supplement: Additional file 2: — Questionnaire. Additional file descriptions text (including details of how to view the file, if it is in a non-standard format). (PDF 130 kb) [file 12879_2015_1237_MOESM2_ESM.pdf]

SOCIAL CONNECTEDNESS STUDY - QUESTIONNAIRE

Your household

H1 Please list in the table all the people who usually live in your household:

	First name + initial	Gender (circle one)	Age (years)
**Person 1 (you)**		Female/Male	
**Person 2**		Female/Male	
**Person 3**		Female/Male	
**Person 4**		Female/Male	
**Person 5**		Female/Male	
**Person 6**		Female/Male	
**Person 7**		Female/Male	
**Person 8**		Female/Male	

H2 What is your current marital status (please circle one)?

Never married	Married	Single	Divorced
Separated	Widowed	Living with partner	

H3 What country were you born in? ____________________

H4 How long have you been in Australia? ____________________years

H5 What is the highest level of education you have completed, either in Australia or overseas? If overseas, please specify which country you were educated in.

	Australia	Overseas (specify)
Primary school	☐	☐
Up to year 10	☐	☐
Year 11	☐	☐
Year 12	☐	☐
TAFE Diploma or business college	☐	☐
University	☐	☐
Other (please specify)	☐	☐

If you are living with a partner:

H3A What country was your partner born in? ________________________

H4A How long has your partner been in Australia? ________________years

H5A What is the highest level of education your partner has completed, either in Australia or overseas? If overseas, please specify which country they were educated in.

	**Australia**	**Overseas (specify)**
Primary school	☐	☐
Up to year 10	☐	☐
Year 11	☐	☐
Year 12	☐	☐
TAFE Diploma or business college	☐	☐
University	☐	☐
Other (please specify)	☐	☐

H6 What languages do you speak at home (please list the main one first)?

1.________________ 2. ________________ 3. ________________

H7 Is your home (please circle one):

Owned outright	Owned with a mortgage	Being purchased under a rent/buy scheme
Being rented	Being occupied rent free	Public Housing
Other (please specify)		

H8 Which of these categories contains the combined income of your household (family members living with you) before tax and other deductions are taken out during the last financial year (1 July 2010 to 30 June 2011)?

Per Week	Per Year	
$2,000 or more	$104,000 or more	☐
$1,600-$1,999	$83,200-$103,999	☐
$1,300-$1,599	$67,600-$83,199	☐
$1,000-$1,299	$52,000-$67,599	☐
$800-$999	$41,600-$51,999	☐
$600-$799	$31,200-$41,599	☐
$400-$599	$20,800-$31,199	☐
$250-$399	$13,000-$20,799	☐
$150-$249	$7,800-$12,999	☐
$1-$149	$1-$7,799	☐
Nil income	Nil income	☐

Your neighbourhood

N1 How long have you lived at this address? ________________years

N2 Why did you come to live here (please circle all that apply)?

Cost of housing	Friends and family lived here	Employment reasons
Liked the area	Government allocated the house	No other housing available
Other (specify)		

N3 Where did you live before you came to this address (please circle one)?

This general area	Some other area

N4 What do you like about living in your neighbourhood (please circle any that apply)?

Family, friends, a sense of belonging	Convenient to shops, services, facilities	Access to public transport
Quiet and pleasant neighbourhood	Quality of local services, facilities	Multicultural neighbourhood
Aspects of housing (affordability, type)	Parks and open spaces	Safe and healthy environment
Other factors (specify)		

N5 Is there anything you dislike about living in your neighbourhood?

____

____

____

____

____

____

Please circle one response to rate the following aspects of your neighbourhood:

N6 How would you rate your neighbourhood as a place to live?

Good	Average	Poor	Don't know

N7 How would you rate local community services?

Good	Average	Poor	Don't know

N8 How would you rate services for families and young children?

Good	Average	Poor	Don't know

N9 How would you rate local health and welfare services?

Good	Average	Poor	Don't know

N10 How would you rate access to recreational and leisure facilities?

Good	Average	Poor	Don't know

N11 How would you rate crime and personal safety in your neighbourhood?

Good	Average	Poor	Don't know

How do you feel about the following crime and safety issues (please circle one response)?

N12 I feel safe walking alone down my street

Disagree strongly	Disagree	Neither	Agree	Agree strongly	Don't know

N13 Children can safely play outside in my neighbourhood

Disagree strongly	Disagree	Neither	Agree	Agree strongly	Don't know

N14 I can trust most people in this neighbourhood

Disagree strongly	Disagree	Neither	Agree	Agree strongly	Don't know

N15 Neighbours around here generally look out for each other

Disagree strongly	Disagree	Neither	Agree	Agree strongly	Don't know

Your connections

How do you feel about the following statements (please circle one response)?

C1 I know quite a few people who live in this neighbourhood

Disagree strongly	Disagree	Neither	Agree	Agree strongly	Don't know

C2 I feel a sense of belonging to this community

Disagree strongly	Disagree	Neither	Agree	Agree strongly	Don't know

C3 Many of my family and friends live in this neighbourhood or close by

Disagree strongly	Disagree	Neither	Agree	Agree strongly	Don't know

C4 I feel generally valued by the community

Disagree strongly	Disagree	Neither	Agree	Agree strongly	Don't know

C5 I feel I have some influence or control over decisions made in this neighbourhood

Disagree strongly	Disagree	Neither	Agree	Agree strongly	Don't know

C6 In an emergency, I could raise $2,000 within 2 days from my relatives and friends

Disagree strongly	Disagree	Neither	Agree	Agree strongly	Don't know

Over the past year, how often have you:

C7 Done voluntary work with a community organization?

Yes, Often	Yes, a few times	Never	Don't know

C8 Visited friends locally?

Yes, Often	Yes, a few times	Never	Don't know

C9 Spoken to your neighbours?

Yes, Often	Yes, a few times	Never	Don't know

C10 Minded a friend's or neighbour's child?

Yes, Often	Yes, a few times	Never	Don't know

C11 Taken part in a local church, sporting or social club?

Yes, Often	Yes, a few times	Never	Don't know

C12 Been out to a local café, pub or show?

Yes, Often	Yes, a few times	Never	Don't know

C13 Been to a public meeting or signed a petition?

Yes, Often	Yes, a few times	Never	Don't know

Your electronic connections

E1 Does your household have:

Internet access	Cable TV	Satellite TV

E2 Which of the following forms of electronic communication do you use?

Email	Video calling<br>eg Skype
Social networking<br>eg Facebook	Blogging<br>eg Twitter
Other (specify)	

E3 If you answered yes to any of the above forms of communication, who do you use them to communicate with?

Family overseas	Family in Australia
Friends in Australia	Friends overseas

Connections and information

I1 If you had a concern about your health, where would you seek information?

1. ____________________

2. ____________________

3. ____________________

4. ____________________

5. ____________________
