# Supplementary material for: Social encounter profiles of greater Melbourne residents, by location – a telephone survey
Source: BMC Infect Dis. 2015 Nov 2;15:494. doi: 10.1186/s12879-015-1237-9 (PMC4631075; doi:10.1186/s12879-015-1237-9)
Supplement: Additional file 3: — Contact Diary. Additional file descriptions text (including details of how to view the file, if it is in a non-standard format). (PDF 61 kb) [file 12879_2015_1237_MOESM3_ESM.pdf]

SAMPLE DIARY CARD MONDAY **SUBJECT ID ☐☐☐**

TIME	AM/PM	LOCATION (DESCRIBE - INCLUDING STREET ADDRESS)	
APPROXIMATE NUMBER OF PEOPLE IN THIS LOCATION WITH YOU			
APPROXIMATE NUMBER OF PEOPLE AT ARMS' LENGTH AT THIS LOCATION			
IF TIME/LOCATION DETAILS ARE AS PREVIOUS PAGE PLEASE TICK HERE			☐

First Name & Initial (if known) OR Unique identifier	Occupation (if known)	Home Suburb (if known)	Age OR Range	M/F	Any physical contact? (Y/N)	Contact Duration (approx)	
						Hrs	Mins
