# Supplementary material for: Social encounter profiles of greater Melbourne residents, by location – a telephone survey
Source: BMC Infect Dis. 2015 Nov 2;15:494. doi: 10.1186/s12879-015-1237-9 (PMC4631075; doi:10.1186/s12879-015-1237-9)
Supplement: Additional file 4: — Additional Results. Additional results including details on number and duration of physical contact encounters, and duration of encounters for each household size by contact type (known/unknown) and location type (home/outside). (PDF 1275 kb) [file 12879_2015_1237_MOESM4_ESM.pdf]

Additional file 4: Additional Results

Contents

1.1 Statistical Methodology 1
1.2 Number of Physical Encounters with Listed People 2
1.3 Number of Unique Contacts 3
1.4 Age-Age Mixing of Listed People by Gender: Number of Encounters (Unweighted) 4
1.5 Age-Age Mixing of Listed People by Gender: Total Duration of Encounters (Unweighted). 4
1.6 Cell Counts for Age-Age Mixing of Listed People: Number of Encounters 5
1.7 Age-Age Mixing of Listed People by Gender: Number of Physical Encounters 6
1.8 Cell Counts for Age-Age Mixing of Listed People: Number of Physical Encounters 7
1.9 Age-Age Mixing of Listed People by Gender: Total Duration of Physical Encounters 8
1.10 Known vs. Unknown Contacts 9
1.11 Age-Age Mixing with Groups by Gender: Number of Encounters 10
1.12 Age-Age Mixing with Groups by Gender: Person-Contact_Hours of Encounters 11

1.1 Statistical Methodology

All results are computed using Stata 10 or Stata 13. Weighted results use weights found by raking, as described in the main paper and Supplementary Material 3.The confidence intervals in the mixing matrices use a t-distribution, where the degrees of freedom accounts for the weighted number of participants, and the robust/sandwich estimator of variance. Weighted boxplots use the weights to determine percentiles.

1.2 Number of Physical Encounters with Listed People

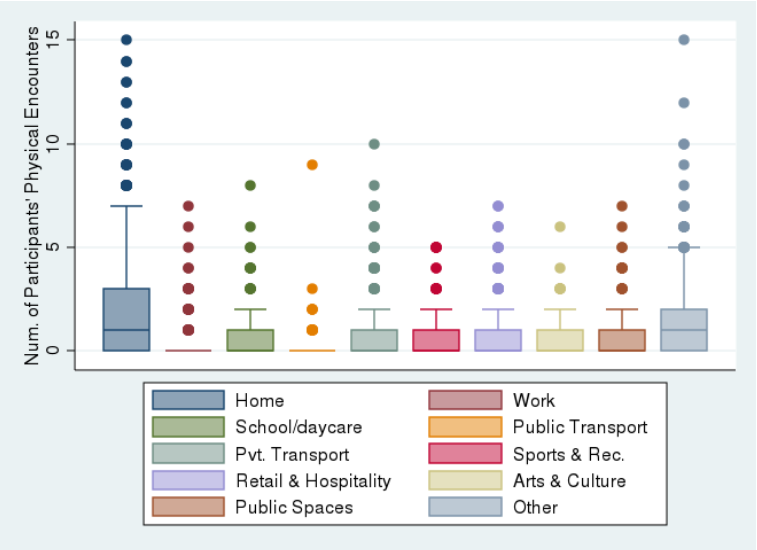

Figure 1 Participants' weighted total number of physical encounters with listed people by location type. Data for each location type are for all participants reporting at least one visit to an address of that location type. For each participant, the numbers of all reported encounters involving physical contact with individually listed people are totalled. Boxes denote the interquartile range, interior lines show the median and the whiskers show adjacent values. Raking weights are used to reduce the effects of sample bias.

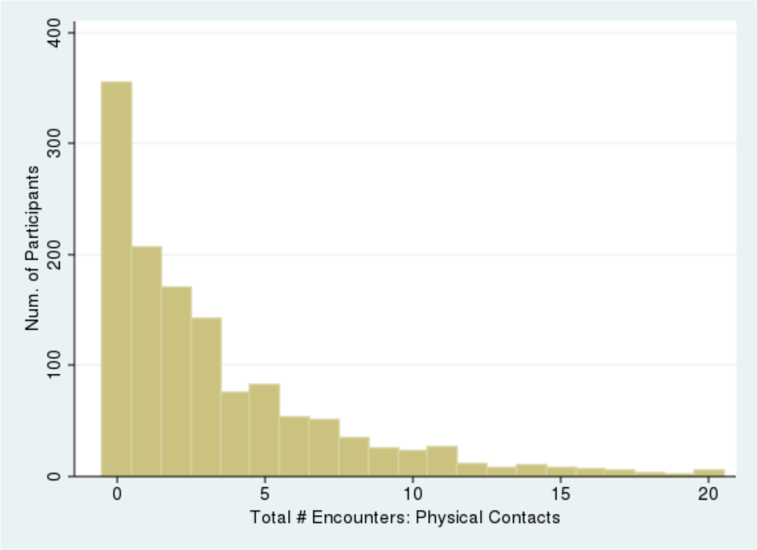

Figure 2 Participants' weighted total number of listed physical encounters. This histogram illustrates the distribution of the total number of encounters involving physical contact with individually listed people reported by each participant**.**

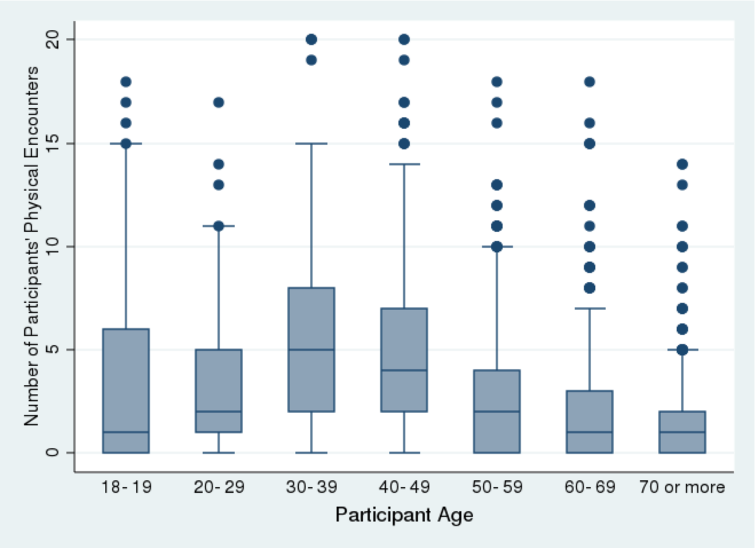

Figure 3 Participants' weighted total number of listed physical encounters by age. This boxplot illustrate the total number of encounters involving physical contact with individually listed people reported by each participant. Results are shown separately by participant age. Raking weights are used to reduce the effects of sample bias.

1.3 Number of Unique Contacts

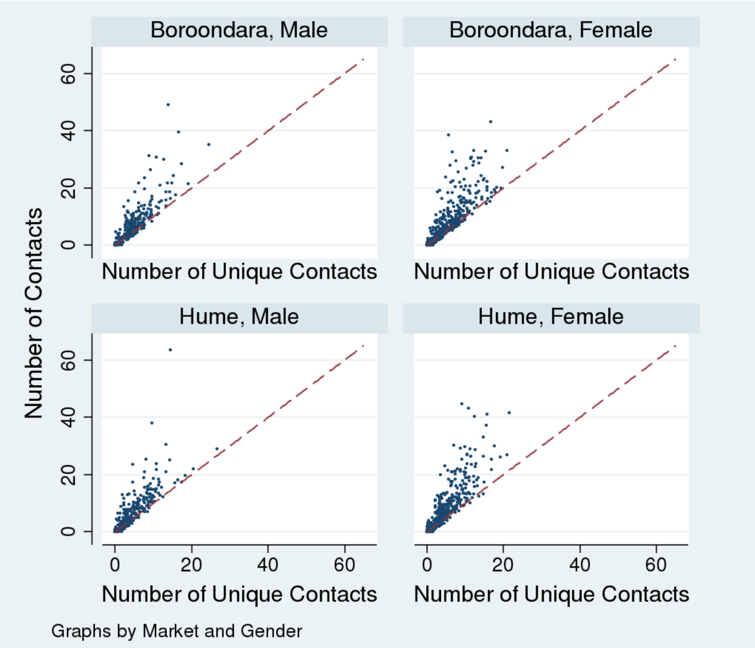

Figure 4 Jittered scatterplot for participants' number of unique contacts vs. all contacts. These scatterplots illustrate the number of unique contacts and contacts reported by each participant, as ordered pairs, separated by city (Boroondara-top row; Hume- bottom row) and gender (male-left column; female-right column) of the participant. The points are jittered to visually separate them slightly since they occur on integers. Note that for each participant, the number of contacts cannot be smaller than the number of unique contacts. Points above the diagonal line illustrate the extent of repeated contacts with individuals.

1.4 Age-Age Mixing of Listed People by Gender: Number of Encounters (Unweighted)

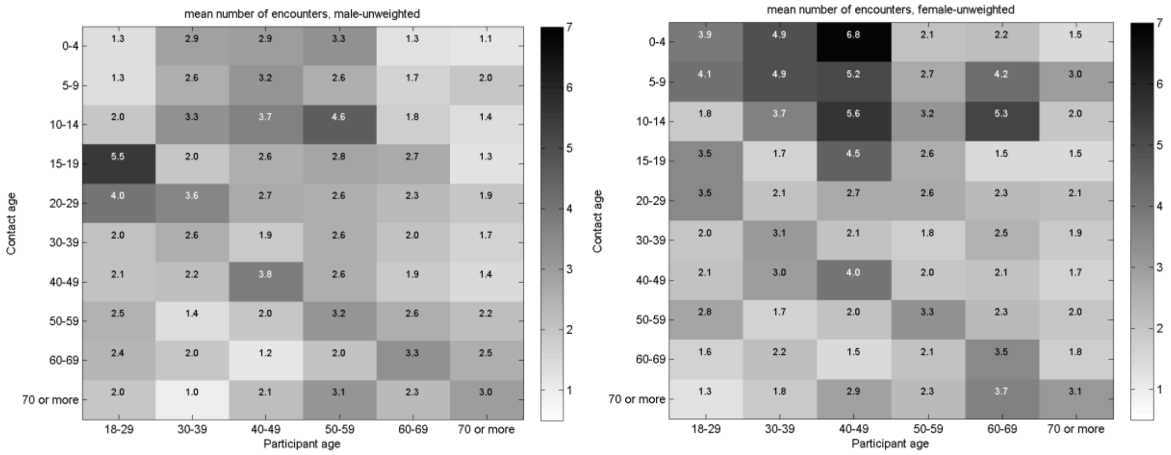

Figure 5 Age-based mixing matrices for participants' total number of encounters with listed individuals. The figures show the mean number of encounters for male (left panel) and female (right panel) participants by participant and contact age groups. Darker shading indicates larger values.

1.5 Age-Age Mixing of Listed People by Gender: Total Duration of Encounters (Unweighted)

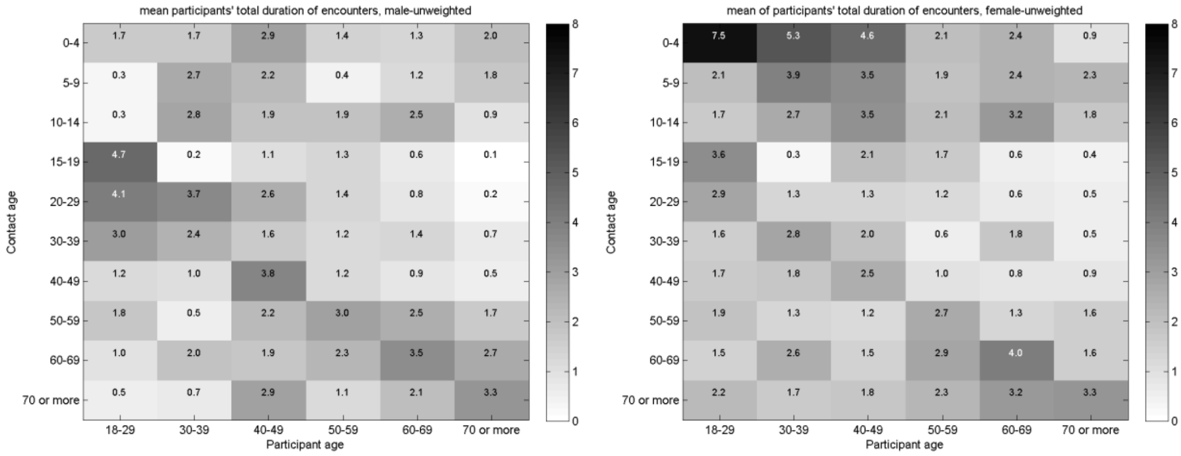

Figure 6 Age-based mixing matrices for participants' total duration of encounters with listed individuals. The figures show the mean duration of encounters for male (left panel) and female (right panel) participants by participant and contact age groups. Darker shading indicates larger values.

1.6 Cell Counts for Age-Age Mixing of Listed People: Number of Encounters

	All Encounters- Male Participants, Unweighted						
	Participant Age						
Contact Age	18- 29	30- 39	40- 49	50- 59	60- 69	70 or more	Refused
0-4	3	9	18	9	14	8	0
5-9	3	7	23	9	3	4	0
10-14	7	4	41	15	5	8	0
15-19	29	2	31	37	10	12	0
20-29	37	9	26	70	55	37	0
30-39	22	19	36	35	52	35	0
40-49	19	9	62	54	39	41	1
50-59	26	8	30	77	61	32	0
60-69	8	5	12	21	71	35	0
70 or more	2	3	7	14	19	73	0
Maximum Possible for Participant Age	56	23	79	112	123	120	1

Table 1 Number of Participants for Age-Age Mixing Matrices (All encounters, Male participants, Unweighted)
(See Figure 5 left panel (above) for the corresponding mixing matrix.)

	All Encounters- Male Participants, Weighted						
	Participant Age						
Contact Age	18- 29	30- 39	40- 49	50- 59	60- 69	70 or more	Refused
0-4	8.09	25.25	43.79	7.69	9.78	3.19	0
5-9	2.74	19.69	57.34	10.27	1.34	2.36	0
10-14	16.23	9.57	92.80	10.30	3.95	3.40	0
15-19	49.87	4.10	63.87	32.63	4.24	6.62	0
20-29	100.17	15.19	52.99	61.50	33.64	15.61	0
30-39	65.49	50.45	86.40	32.34	32.20	17.54	0
40-49	41.57	22.14	143.19	42.82	27.06	20.62	1.75
50-59	54.98	19.95	75.33	71.96	37.91	13.11	0
60-69	9.94	6.37	30.18	17.81	38.09	15.54	0
70 or more	3.02	3.12	13.61	11.84	10.58	32.29	0
Maximum Possible for Participant Age	131.20	57.01	188.53	109.76	76.44	60.83	1.75

Table 2 Number of Participants for Age-Age Mixing Matrices (All encounters, Male participants, Weighted)
(See Main paper, Figure 4 left panel for the corresponding mixing matrix.)

	All Encounters- Female Participants, Unweighted						
	Participant Age						
Contact Age	18- 29	30- 39	40- 49	50- 59	60- 69	70 or more	Refused
0-4	9	33	12	18	22	8	0
5-9	7	38	39	14	14	9	0
10-14	6	16	61	18	8	12	0
15-19	30	12	54	52	14	19	0
20-29	44	29	55	115	75	49	0
30-39	14	54	48	90	75	43	0
40-49	27	31	84	80	66	72	0
50-59	36	20	50	112	63	73	0
60-69	11	16	23	60	95	54	0
70 or more	4	6	12	37	47	105	0
Maximum Possible for Participant Age	69	67	114	181	167	194	1

Table 3 Number of Participants for Age-Age Mixing Matrices (All encounters, Female participants, Unweighted)
(See Figure 5 right panel (above) for the corresponding mixing matrix.)

	All Encounters- Female Participants, Weighted						
	Participant Age						
Contact Age	18- 29	30- 39	40- 49	50- 59	60- 69	70 or more	Refused
0-4	24.61	56.08	13.74	9.11	11.03	2.77	0.00
5-9	16.45	51.84	48.40	7.56	6.60	3.74	0.00
10-14	2.72	20.67	76.57	7.25	3.94	4.54	0.00
15-19	44.91	15.25	68.52	30.53	6.07	8.17	0.00
20-29	85.52	39.40	76.22	76.40	33.91	18.88	0.00
30-39	55.88	84.35	62.26	61.52	34.58	18.65	0.00
40-49	46.36	47.04	107.43	55.57	29.97	31.36	0.00
50-59	55.43	25.23	72.27	75.72	28.52	33.30	0.00
60-69	22.68	21.29	35.13	36.78	42.00	24.97	0.00
70 or more	4.41	5.34	12.25	19.79	23.02	43.52	0.00
Maximum Possible for Participant Age	140.49	99.12	158.2	117.25	80.27	85.65	0.40

Table 4 Number of Participants for Age-Age Mixing Matrices (All encounters, Female participants, Weighted)
(See Main paper, Figure 4 right panel for the corresponding mixing matrix.)

1.7 Age-Age Mixing of Listed People by Gender: Number of Physical Encounters

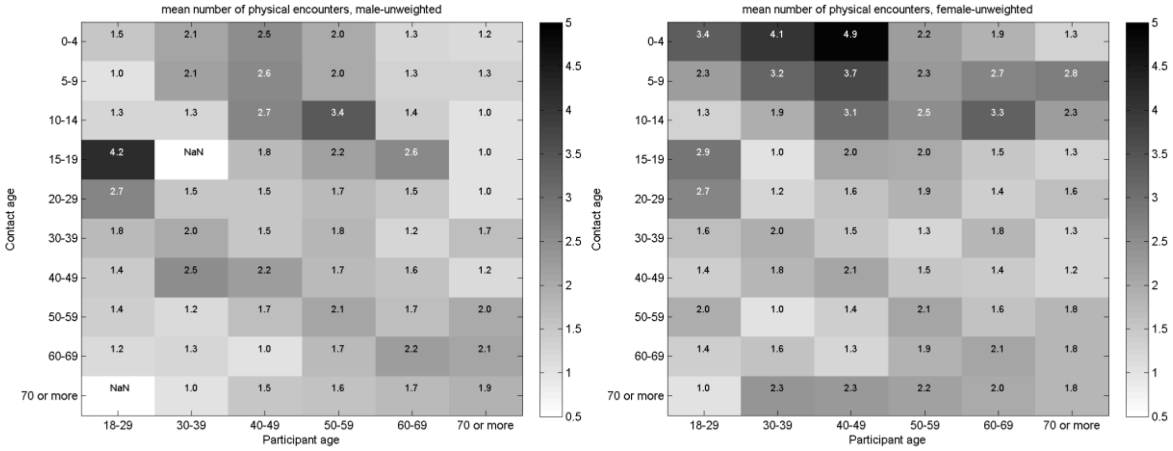

Figure 7 Age-based mixing matrices for participants' total number of physical encounters with listed individuals. The figures show the mean number of physical encounters for male (left panel) and female (right panel) participants by participant and contact age groups. Darker shading indicates larger values.

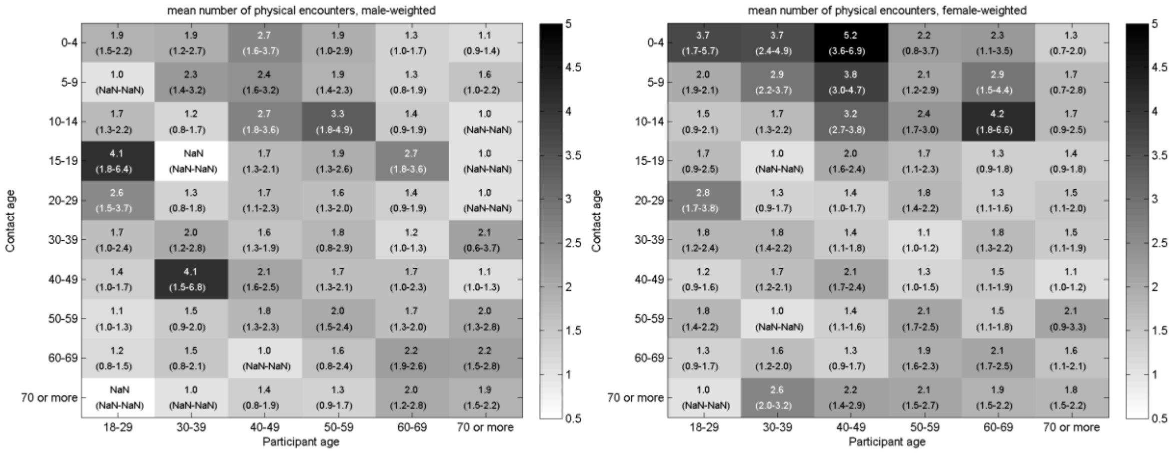

Figure 8 Age-based mixing matrices for participants' weighted total number of physical encounters with listed individuals. The figures show the weighted mean (over participants) number of physical encounters for male (left panel) and female (right panel) participants by participant and contact age groups. 95% confidence intervals are shown in parentheses.. Darker shading indicates larger values. Women notably have more numerous physical contact with children less than 15 years old. Raking weights are used to reduce the effects of sample bias.

1.8 Cell Counts for Age-Age Mixing of Listed People: Number of Physical Encounters

Physical Encounters- Male Participants, Unweighted							
	Participant Age						
Contact Age	18- 29	30- 39	40- 49	50- 59	60- 69	70 or more	Refused
0-4	2	9	14	5	11	6	0
5-9	1	7	21	8	3	3	0
10-14	4	4	31	10	5	3	0
15-19	15	0	17	17	5	1	0
20-29	20	6	11	31	16	5	0
30-39	4	13	12	11	23	9	0
40-49	9	4	47	28	12	12	0
50-59	14	6	21	57	38	16	0
60-69	5	4	5	10	50	21	0
70 or more	0	2	4	5	11	46	0
Maximum Possible for Participant Age	56	23	79	112	123	120	1

Table 5 Number of Participants for Age-Age Mixing Matrices (Physical encounters, Male participants, Unweighted)
(See Figure 7 left panel (above) for the corresponding mixing matrix.)

Physical Encounters- Male Participants, Weighted							
	Participant Age						
Contact Age	18- 29	30- 39	40- 49	50- 59	60- 69	70 or more	Refused
0-4	6.94	25.25	36.58	5.15	7.84	2.87	0
5-9	1.01	19.69	53.69	10.09	1.34	1.97	0
10-14	8.71	9.57	67.91	5.88	3.95	1.70	0
15-19	26.94		33.47	11.70	2.25	0.44	0
20-29	58.29	8.65	19.97	22.47	9.36	1.51	0
30-39	14.83	36.21	22.35	9.94	15.69	4.84	0
40-49	10.47	12.78	109.38	23.07	6.50	5.16	0
50-59	38.18	15.84	47.00	54.71	19.51	6.53	0
60-69	5.84	6.28	8.34	9.31	24.26	9.07	0
70 or more		3.03	10.51	4.61	6.35	19.39	0
Maximum Possible for Participant Age	131.20	57.01	188.53	109.76	76.44	60.83	1.75

Table 6 Number of Participants for Age-Age Mixing Matrices (Physical encounters, Male participants, Weighted)
(See Figure 8 left panel (above) for the corresponding mixing matrix.)

Physical Encounters- Female Participants, Unweighted							
	Participant Age						
Contact Age	18- 29	30- 39	40- 49	50- 59	60- 69	70 or more	Refused
0-4	8	32	12	14	20	6	0
5-9	4	37	37	13	9	5	0
10-14	3	13	55	13	8	8	0
15-19	15	1	41	26	4	7	0
20-29	30	9	16	52	22	8	0
30-39	8	38	16	28	35	11	0
40-49	13	13	60	24	23	25	0
50-59	26	6	24	64	30	30	0
60-69	5	7	8	35	65	16	0
70 or more	1	3	10	19	26	57	0
Maximum Possible for Participant Age	69	67	114	181	167	194	1

Table 7 Number of Participants for Age-Age Mixing Matrices (Physical encounters, Female participants, Unweighted)
(See Figure 7 right panel (above) for the corresponding mixing matrix.)

Physical Encounters- Female Participants, Weighted							
	Participant Age						
Contact Age	18- 29	30- 39	40- 49	50- 59	60- 69	70 or more	Refused
0-4	24.29	52.88	13.74	6.89	10.01	2.05	0
5-9	13.13	49.90	45.78	6.34	4.21	1.82	0
10-14	1.36	16.36	67.73	5.42	3.94	2.98	0
15-19	22.21	1.31	45.50	16.59	1.92	3.49	0
20-29	63.06	15.32	23.43	29.13	9.52	2.75	0
30-39	38.02	65.61	17.17	18.85	15.79	6.52	0
40-49	18.54	17.39	72.02	17.87	10.53	11.87	0
50-59	40.13	9.78	33.58	40.95	15.49	15.42	0
60-69	6.46	6.16	14.67	20.81	28.03	8.11	0
70 or more	0.69	1.85	9.80	7.81	11.89	22.62	0
Maximum Possible for Participant Age	140.49	99.12	158.2	117.25	80.27	85.65	0.40

Table 8 Number of Participants for Age-Age Mixing Matrices (Physical encounters, Female participants, Weighted)
(See Figure 8, right panel (above) for the corresponding mixing matrix.)

1.9 Age-Age Mixing of Listed People by Gender: Total Duration of Physical Encounters

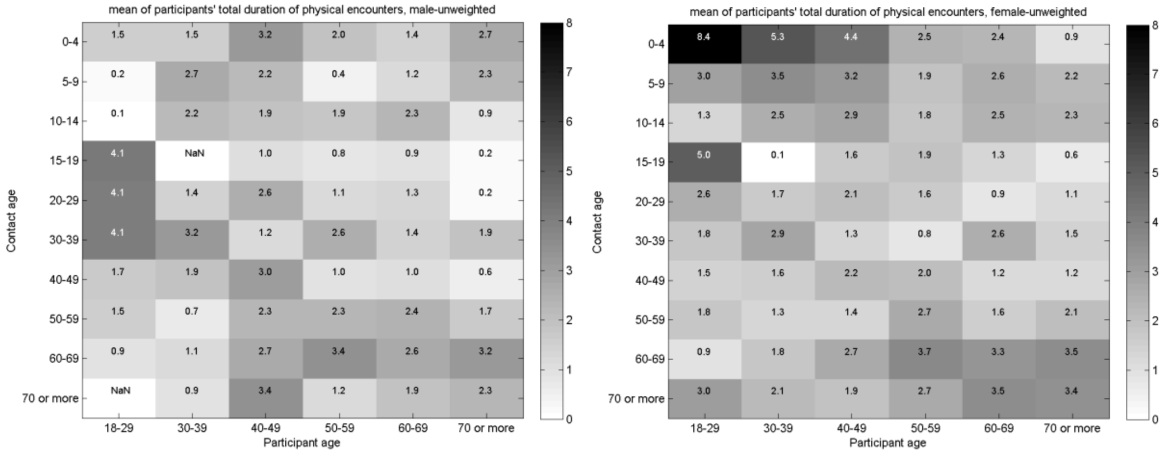

Figure 9 Age-based mixing matrices for participants' total duration of physical encounters with listed individuals. The figures show the mean duration of physical encounters for male (left panel) and female (right panel) participants by participant and contact age groups. Darker shading indicates larger values.

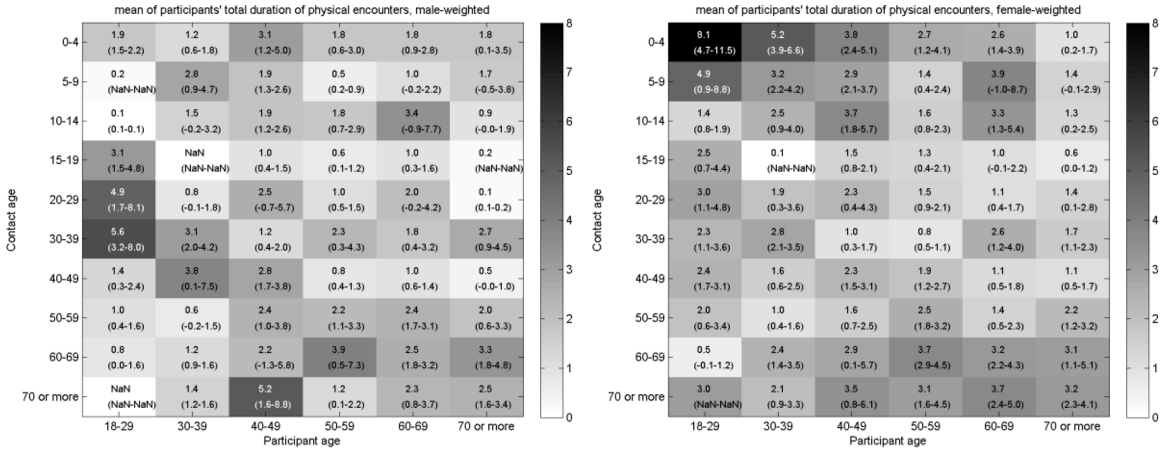

Figure 10 Age-based mixing matrices for participants' weighted total duration of physical encounters with listed individuals. The figures show the weighted mean (over participants) duration of physical encounters for male (left panel) and female (right panel) participants by participant and contact age groups. 95% confidence intervals are shown in parentheses. Darker shading indicates larger values. While age-based assortative mixing is evident, the large duration of physical contact between women and pre-school age children is most notable. Raking weights are used to reduce the effects of sample bias.

1.10 Known vs. Unknown Contacts

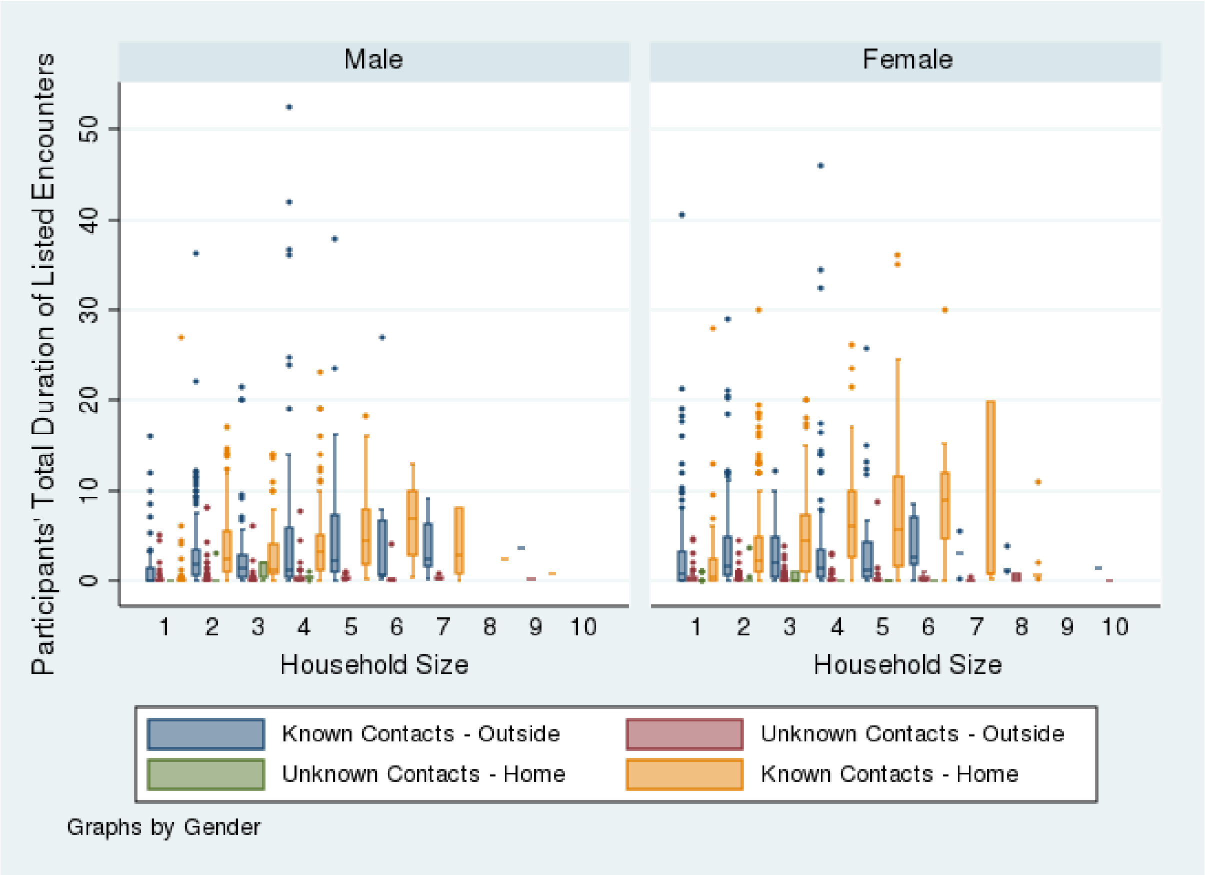

Figure 11 Participants' weighted total duration of listed encounters by household size. Data for the figure are the duration of encounters with individually listed people, totalled for each participant. Contact people are distinguished as "Known" and "Unknown". Contact locations are distinguished as within the home ("Home") or outside the home ("Outside"). Results are reported based on the number of people living in the participant's household (household size), and reported separately for male (left panel) and female (right panel) participants. Although a large amount of heterogeneity is evident, the duration of contact with known individuals generally increases with household size. Raking weights are used to reduce the effects of sample bias.

1.11 Age-Age Mixing with Groups by Gender: Number of Encounters

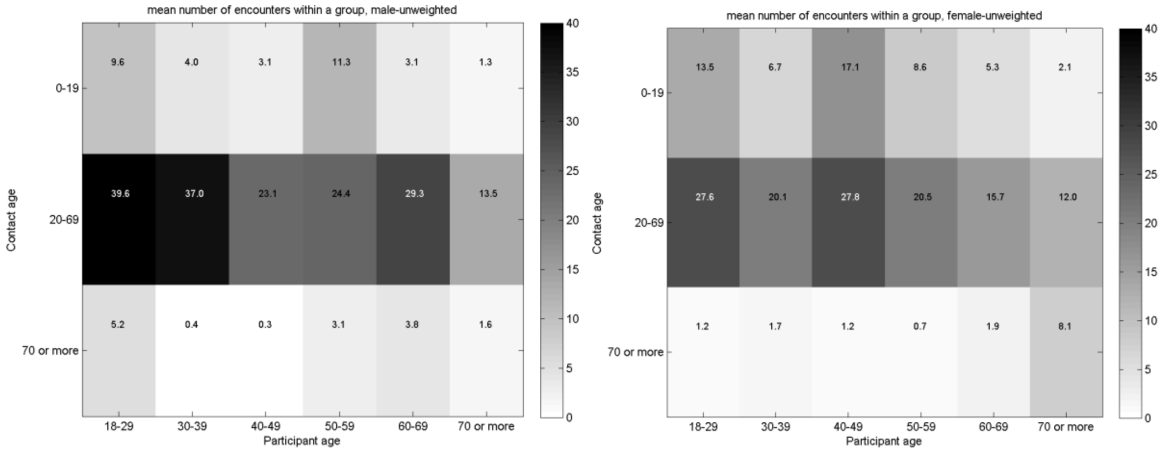

Figure 12 Age-based mixing matrices for number of encounters within groups. The figures show the mean (over participants) number of encounters within a group for male (left panel) and female (right panel) participants by age of the participant and the typical age of group members. Each group was Darker shading indicates larger values.

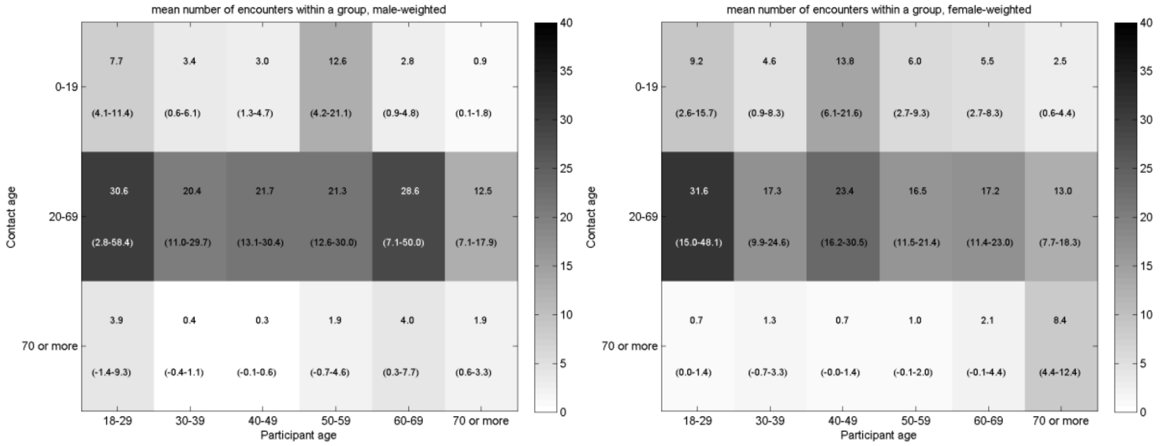

Figure 13 Age-based mixing matrices for number of encounters within groups (weighted). The figures show the weighted mean (over participants) number of encounters within a group for male (left panel) and female (right panel) participants by age of the participant and the typical age of group members. 95% confidence intervals are shown in parentheses. Darker shading indicates larger values. Raking weights are used to reduce the effects of sample bias.

1.12 Age-Age Mixing with Groups by Gender: Person-Contact_Hours of Encounters

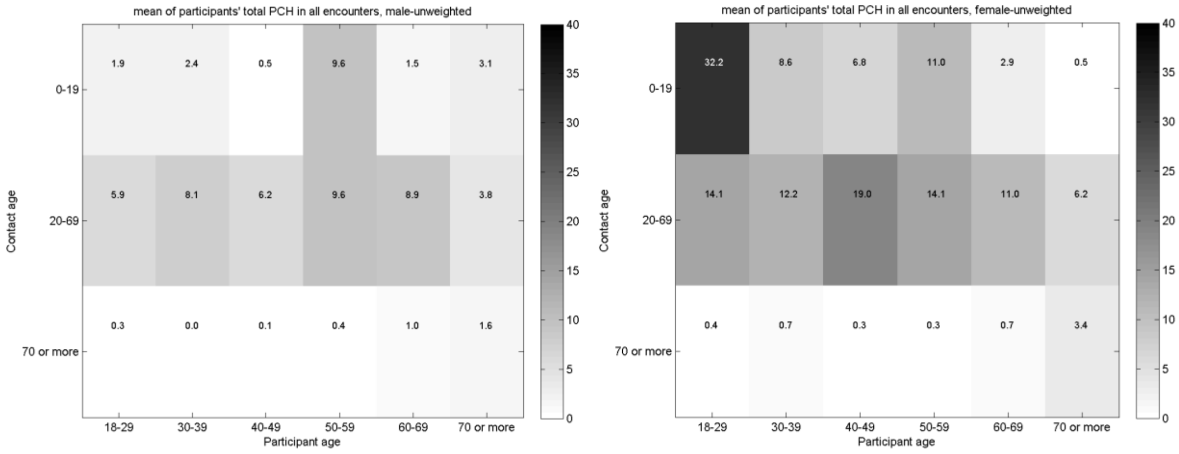

Figure 14 Age-based mixing matrices for person-contact-hours of encounters within groups. The figures show the mean (over participants) person-contact-hours of encounters within a group for male (left panel) and female (right panel) participants by age of the participant and the typical age of group members. Darker shading indicates larger values.

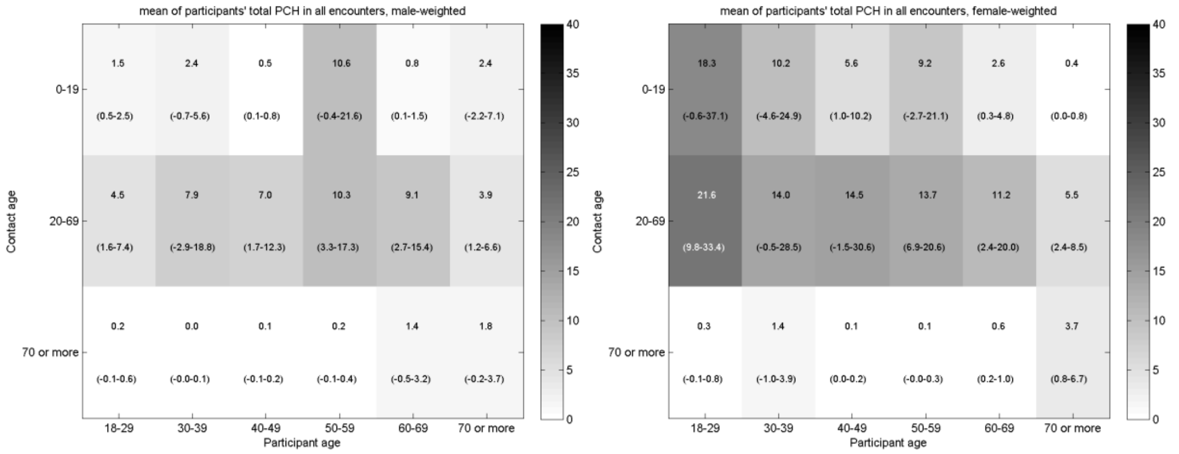

Figure 15 Age-based mixing matrices for person-contact-hours of encounters within groups (weighted). The figures show the weighted mean (over participants) person-contact-hours of encounters within a group for male (left panel) and female (right panel) participants by age of the participant and the typical age of group members. 95% confidence intervals are shown in parentheses. Darker shading indicates larger values. Differences in contact patterns between men and women are apparent, particularly for participants aged 18 to 49. Raking weights are used to reduce the effects of sample bias.
